# CD137 agonists in mice suppress germinal center responses by increasing the immunosuppressive activity of follicular regulatory T-cells

**DOI:** 10.1038/s44318-026-00900-2

**Published:** 2026-08-25

**Authors:** Ana Martínez-Riaño, Raquel Cuesta, Dayanna Salinas, Nekane Soria, Raluca Alexandru, Paula Molero-Glez, David Ruiz-Guillamon, Arantza Azpilikueta, Carlos Luri-Rey, Álvaro Lopez-Janeiro, Carlos E de Andrea, María S Aymerich, Pedro Berraondo, Álvaro Teijeira, Ignacio Melero

**Affiliations:** 1 Program of Immunology and Immunotherapy, Cima Universidad de Navarra, Cancer Center Clínica Universidad de Navarra (CCUN), Pamplona, Spain; 2 Biogipuzkoa, Oncology department, 20014 San Sebastián, Spain; 3 Department of Pathology, Clínica Universidad de Navarra (CCUN), Pamplona, Spain; 4 Centro de Investigación Médica Aplicada, Pamplona, Spain; 5 Navarra Institute for Health Research (IDISNA), Pamplona, Spain; 6 Centro de Investigación Biomédica en Red de Cáncer (CIBERONC), Madrid, Spain; 7 Nuffield Department of Medicine. University of Oxford, Oxford, UK

**Keywords:** Immunology

## Abstract

Humoral immune responses rely on germinal center (GC) reactions to generate long-lasting antibody responses with affinity maturation. CD137 (4-1BB) agonists are known to exert inhibitory effects on B-cell responses; however, the underlying mechanisms remain unclear. Here, we show that agonistic anti-CD137 monoclonal antibodies target multiple CD137-expressing cell types, including activated CD8⁺ T-cells, regulatory T-cells, follicular regulatory T-cells, and follicular dendritic cells. Anti-CD137 antibodies strongly disrupt GC reactions upon vaccination, preventing the development of anti-drug antibodies, and suppress ectopic B-cell responses within tumor-associated tertiary lymphoid structures in mice. Mechanistic analyses demonstrate that GC disruption occurs independently of IFN-γ, IL-12, and CD8⁺ T-cells, but is critically dependent on Foxp3⁺ T-cells. Combined transcriptomic profiling, immune phenotyping, and functional GC suppression assays reveal that anti-CD137 treatment increases the abundance and immunosuppressive activity of regulatory and follicular regulatory T-cells in vaccinated mice. Our results uncover the cellular basis of CD137-mediated suppression of humoral immunity and provide insight into rational combinatorial immunotherapeutic strategies incorporating CD137 agonists under active clinical development.

## Synopsis

Agonistic CD137 (4-1BB) antibodies are promising cancer immunotherapies, but their inhibitory effects on humoral immunity remain poorly understood. This study shows that CD137 agonists abolish germinal center (GC) responses by enhancing the suppressive activity of follicular regulatory T-cells, thereby inhibiting B-cell responses after vaccination, in tumor-associated tertiary lymphoid structures, and against therapeutic antibodies.

- A single dose of an agonistic anti-CD137 antibody completely abolishes GC responses after vaccination.
- Agonistic anti-CD137 antibodies bind primarily to activated CD8⁺ T-cells, follicular regulatory T-cells, regulatory T-cells, and follicular dendritic cells.
- The inhibitory effect is independent of IFN-γ, IL-12, and CD8⁺ T-cells, but depends on a radiosensitive hematopoietic compartment.
- GC suppression is mediated, at least in part, by the expansion of Foxp3⁺ CD4⁺ T-cells (regulatory and follicular regulatory T-cells) and their enhanced suppressive activity.
- CD137 agonists also suppress B-cell responses in tumor-associated tertiary lymphoid structures and reduce anti-drug antibody formation.

## Abstract (web summary)

Agonistic anti-CD137 (4-1BB) monoclonal antibodies target multiple cell types and suppress B-cell responses through the expansion of Foxp3⁺ CD4⁺ T-cells.

## Graphical abstract

**Figure:**
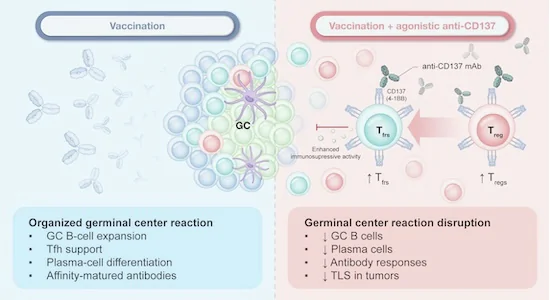

## Introduction

CD137/4-1BB (Tnfrsf9) is a co-stimulatory receptor that belongs to the tumor necrosis factor receptor superfamily (TNFRSF) (Watts et al, 2025), expressed mainly on activated T lymphocytes and NK cells (Kwon et al, 1989; Melero et al, 1998; Wang et al, 2009), but also on activated B cells (Zhang et al, 2010) and on certain myeloid leukocytes, including dendritic cells (Futagawa et al, 2002; Lee et al, 2005; Nishimoto et al, 2005). Its ligand, CD137L (4-1BBL) (Goodwin et al, 1993), is expressed on the surface of antigen-presenting cells such as dendritic cells, macrophages, and B cells (Watts et al, 2025). Engagement of CD137 enhances T-cell proliferation/survival, cytotoxic functions, and increases IL-2 and IFN-γ expression (Otano et al, 2021). Among its multiple roles, CD137 stimulation has been implicated in the prevention/reversion of T-cell exhaustion (Andreata et al, 2024; Molero-Glez et al, 2024). All these functions are mediated by downstream signaling through TRAF and cIAP adapters encompassing K63 polyubiquitination reactions (Glez-Vaz et al, 2023; Zapata et al, 2018).

Owing to these effector functions, agonistic CD137 antibodies have emerged as suitable agents for cancer immunotherapy. Preclinical data show that CD137 agonism improves antitumor responses in transplantable cancer mouse models (Melero et al, 1997). Importantly, CD137 agonists have shown synergistic therapeutic activity in combinations with a variety of anticancer therapeutics, including checkpoint-inhibitor immunotherapy (Melero et al, 2023a). In human cancer therapeutics, there are over a dozen agents under clinical development targeting 4-1BB co-stimulation to tumors with promising safety and efficacy profiles (Melero et al, 2023a; Melero et al, 2023b; Muik et al, 2022; Piha-Paul et al, 2025) that overcome the serious liver safety issues of the first-generation agonist urelumab (Segal et al, 2017), also partially observed in mice (Dubrot et al, 2010).

While the effects of agonistic antibodies on CD8^+^ T cells have been extensively studied, their impact on other subsets, such as regulatory T cells (Tregs), which also express 4-1BB, remains less understood (So et al, 2008), particularly in the context of vaccination. In this regard, CD137 is prominently expressed in tumor-infiltrating Treg cells in mice and humans (Freeman et al, 2020), but the effect of CD137 agonist ligation on these Tregs remains unclear (Kumar et al, 2018).

Following vaccination or infection, the arrival of antigens in secondary lymphoid organs (SLOs) initiates T- and B-cell responses. Activated B cells generate an initial wave of extrafollicular, short-lived plasma cells and memory B cells. Upon receiving co-stimulatory signals from CD4⁺ T-helper cells, a subset of these activated B cells migrates into the inner follicle and initiates a germinal center (GC) response (Victora and Nussenzweig, 2022). Germinal centers are transient structures in which B cells undergo several rounds of somatic hypermutation and affinity maturation (Gitlin et al, 2014; Victora et al, 2010), depending on their ability to acquire antigen from specialized stromal antigen-presenting cells known as follicular dendritic cells (FDCs) (Chen et al, 2023; Martinez-Riano et al, 2023; Suzuki et al, 2009) and to present processed antigens to T follicular helper (Tfh) cells to obtain pro-survival signals in return (Gitlin et al, 2015; Victora et al, 2010). Long-lived, high-affinity plasma cells and memory B cells emerge from GC B cells. This multistep differentiation process is tightly controlled to prevent dysregulation that could facilitate pathogenic mechanisms. Factors such as the persistence and accessibility of antigens to GC B cells, which fuel continuous B-cell proliferation and somatic hypermutation, are stringently controlled (Krimpenfort et al, 2024; Mayer et al, 2020; Singh et al, 2020). Among the regulatory mechanisms in place, follicular regulatory T cells (Tfr) have been shown to facilitate GC shut-down.

Tfr cells share immunosuppressive features with conventional Tregs (including CTLA-4, LAG-3 and TIGIT expression as well as TGF-β and IL10 production). Specifically, Tfr express Neuritin and a differential transcriptional profile with the expression of the transcription factor Bcl6, which is the master regulator of Tfh differentiation (Cavaco et al, 2025; Chung et al, 2011; Gonzalez-Figueroa et al, 2021; Linterman et al, 2011; Wollenberg et al, 2011). Using genetically modified mouse models, Tfr cells have been shown to regulate antibody responses elicited during autoimmunity, allergies, infection, and in response to foreign antigens in vaccines (Botta et al, 2017; Chung et al, 2011; Clement et al, 2019; Fu et al, 2018; Linterman et al, 2011; Wollenberg et al, 2011), especially at early stages of GC formation. Tfrs seem to play a less relevant role at later stages of humoral responses (Clement et al, 2019). Importantly, a spike in Tfr (Foxp3⁺ Bcl6⁺) frequency reportedly precedes GC reaction termination (Jacobsen et al, 2021). Differentiation of Tfr has been shown to be a complex multi-stage process (Lee et al, 2026) where such cells may arise from conventional Tregs (Linterman et al, 2011; Wollenberg et al, 2011) or induced Treg cells (Aloulou et al, 2016). However, some reports also suggest that they can differentiate from Tfh cells at late stages of the GC response (Cavaco et al, 2025; Jacobsen et al, 2021).

Treatment with agonistic CD137 antibodies has been reported to reduce serum autoantibody levels in models of autoimmunity, such as collagen-induced arthritis (Seo et al, 2004) and lupus (Sun et al, 2002; Vinay et al, 2007), as well as during infection, gene-therapy treatment with viral vectors (Quetglas et al, 2012) and vaccination (Hong et al, 2020; Mittler et al, 1999; Sun et al, 2005). CD137 agonists reportedly promote an extrafollicular memory B-cell differentiation pathway in Plasmodium infection (Caloba et al, 2025). However, the underlying cellular and molecular mechanisms acting in the suppression of B-cell responses remain unclear.

Here, we show that in a mouse vaccination model, a single dose of an agonistic anti-CD137 antibody completely abrogates B-cell responses in GCs, independently of the route of immunization and the secondary lymphoid organs analyzed. Using fluorescently labeled anti-CD137 antibodies, we observed preferential binding to Tfrs and FDCs as potential sites of action for suppression of B-cell responses. Although we detected an impaired FDC network expansion, their ability to capture and display antigen was preserved upon treatment with CD137 agonists. Bone marrow reconstitution and adoptive transfer experiments revealed that GC inhibition is dependent on a radiosensitive hemopoietic lineage. Mechanistically, we found that GC suppression was not dependent on IFN-γ, IL-12, or CD8⁺ T cells, but was at least partially dependent on Tregs/Tfrs cells. Notably, immunized mice treated with anti-CD137 agonists exhibited a twofold increase in Treg frequencies with enhanced suppressive activity, along with a disruption of the Tfh/Tfr balance driven by increased Tfr numbers, resulting from both an expansion of Treg precursors and increased Tfr proliferation. Similarly, agonistic CD137 antibodies also reduced B-cell responses in tumor-associated tertiary lymphoid structures (TLS), and diminished anti-drug antibodies (ADAs) in a manner relevant for combined immunotherapy regimens with other monoclonal antibodies (Kohrt et al, 2012; Perez-Gracia et al, 2014).

Our studies reveal the disruption of GC reactions by agonistic anti-CD137 mAbs that markedly inhibit humoral responses. Moreover, we report a role for Tfr expansion in mediating these effects of CD137 agonists on B-cell responses, which are relevant both upon vaccination and for tumor-associated tertiary lymphoid structures.

## Results

### Inhibition of germinal center reactions by agonistic anti-CD137 antibodies

**Figure 1:**
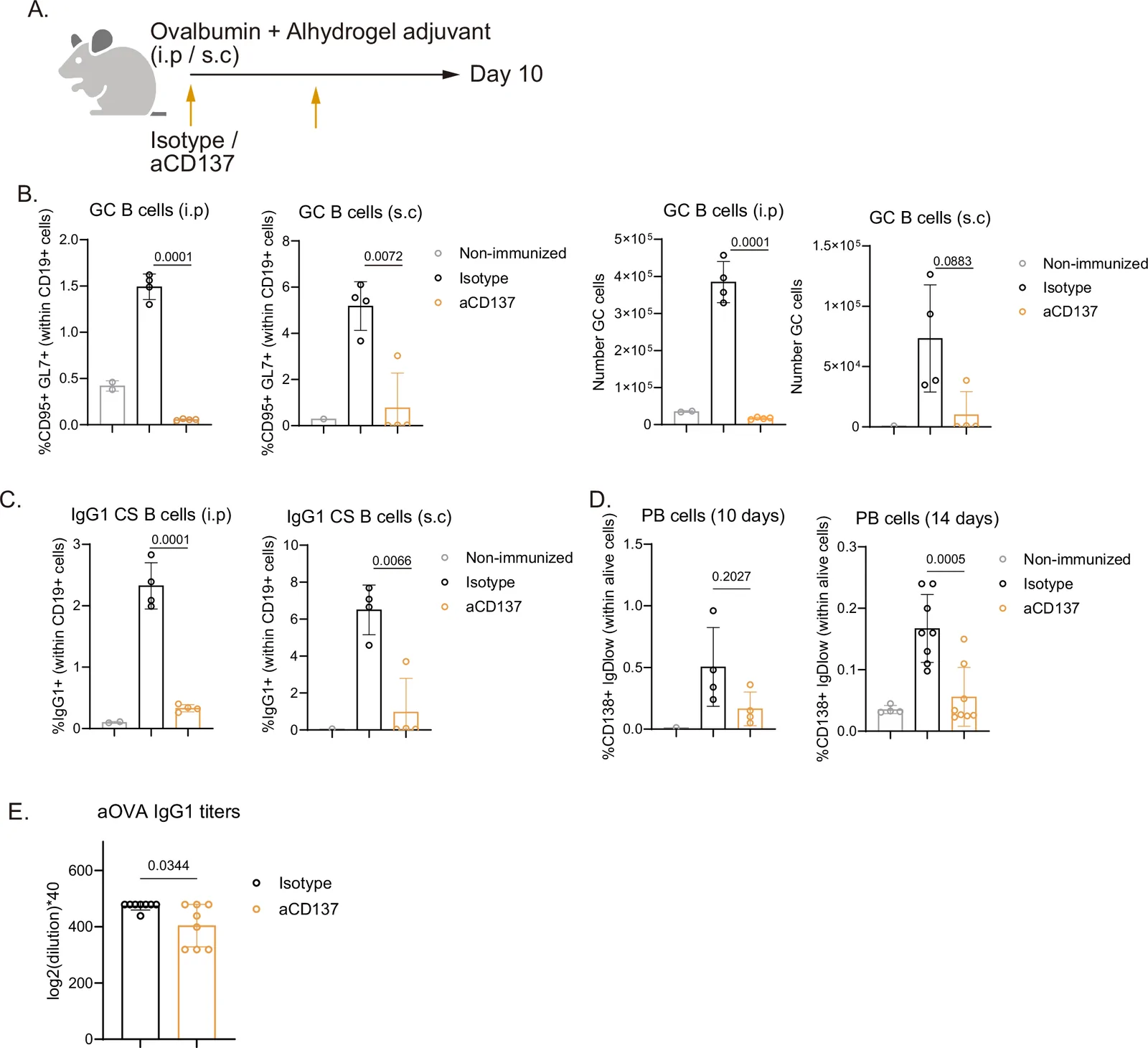
Inhibition of germinal-center responses by anti-CD137 agonistic antibody. (**A**) Immunization workflow used to analyze the effect of 100 μg of the anti-CD137 agonistic antibody (clone 3H3) or its isotype control administered on day 0 or 4 in mice immunized with ovalbumin mixed with Alhydrogel. Mice were immunized intraperitoneally (i.p., 100 μg) or subcutaneously (s.c.; 10 μg per site). Spleens (i.p.) and dLNs (s.c.) were analyzed 10 days following immunization. (**B**) Percentage (left) and total number (right) of GC B cells (CD95^+^ GL7^+^) in non-immunized, intraperitoneally or subcutaneously immunized mice treated with anti-CD137 or isotype control on day 0 (*n* = 4 mice/group; representative of 2 independent experiments). (**C**) Quantification of IgG1^+^ class-switched GC B cells in intraperitoneally or subcutaneously immunized mice treated with anti-CD137 or isotype control (*n* = 4 mice/group; representative of 2 independent experiments). (**D**) Quantification of plasmablasts in the dLNs of mice immunized for 10 (*n* = 4 mice) and 14 days (*n* = 8 mice/group; data pooled from 2 experiments) and treated at day 0 with anti-CD137 or isotype control. (**E**) Anti-ovalbumin IgG1 titers from sera of mice immunized for 14 days with ovalbumin and treated with anti-CD137 or isotype control (*n* = 8 mice/group; data pooled from 2 experiments). All quantitative data show means ± SD and were analyzed using one-way ANOVA tests with multiple comparisons or *t*-test. Source data are available online for this figure.

**Figure EV1:**
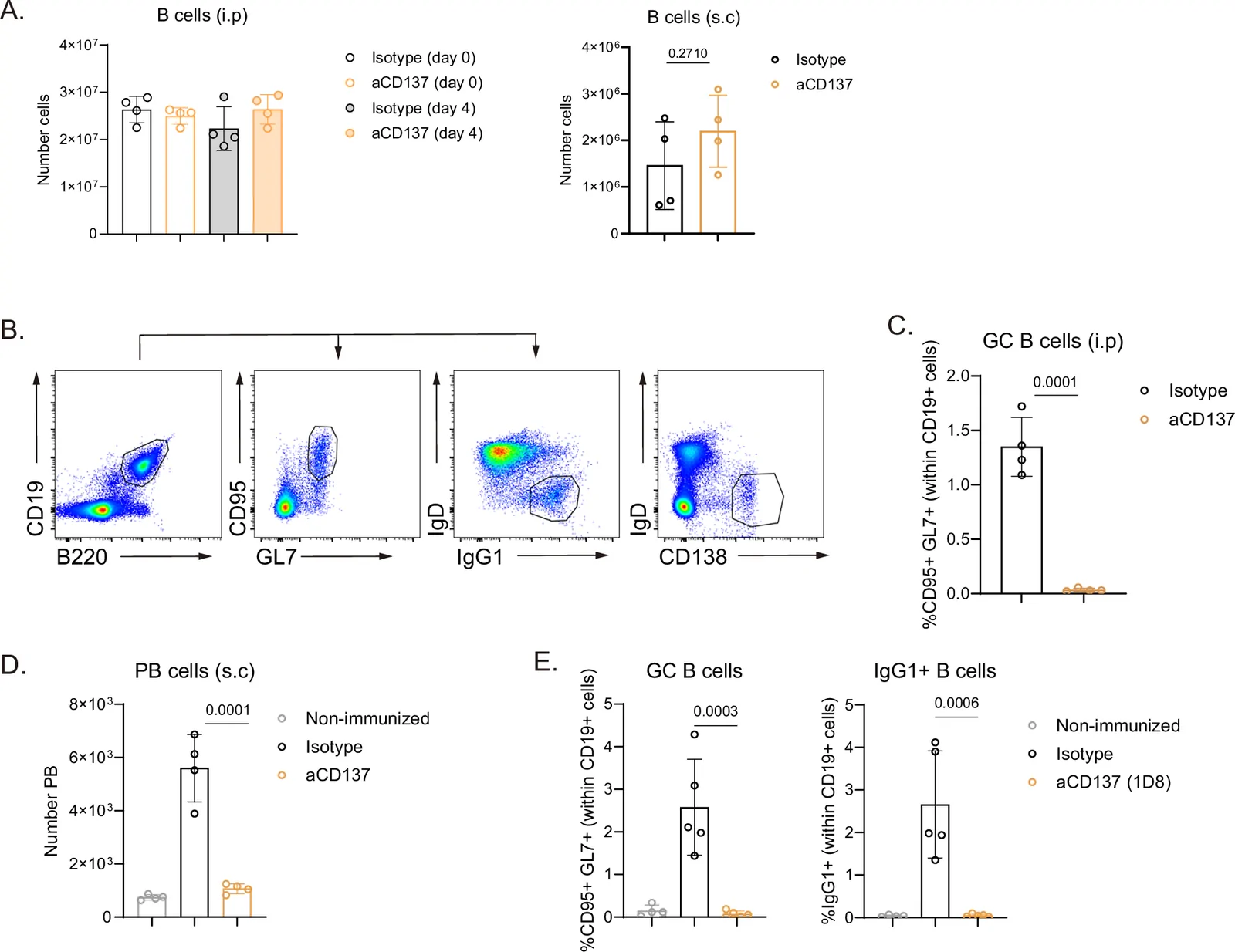
Methodology and analysis of B-cell responses upon anti-CD137 treatment. (**A**) Number of B cells in the spleen (left) or dLNs (right) of mice immunized with ovalbumin mixed with Alhydrogel intraperitoneally (100 µg) or subcutaneously (10 µg/site). Mice immunized intraperitoneally were treated at day 0 or 4 with anti-CD137 or its isotype control (100 µg i.p). Mice immunized subcutaneously were treated at day 0 with anti-CD137 or its isotype control (100 µg i.p) (*n* = 4 mice; representative of two independent experiments). (**B**) Flow cytometry gating strategy for B cells, GC B cells, IgG1 CS B cells and PB. (**C**) Percentage of GC B cells in the spleen of mice immunized i.p with ovalbumin mixed with Alhydrogel and treated at day 4 with anti-CD137 or its isotype control (100 µg i.p) (*n* = 4 mice; representative of two independent experiments). (**D**) Number of PB in the dLNs of non-immunized or immunized mice treated at day 0 with anti-CD137 or its isotype control (100 µg i.p) (*n* = 4 mice). (**E**) Percentage of GC B cells (left graph) and IgG1+ B cells (right graph) in non-immunized or immunized mice treated with isotype or anti-CD137 1D8 mAb (*n* = 5 mice; 1 independent experiment). All quantitative data were shown as means ± SD and analyzed using one-way ANOVA with multiple comparisons or *t*-tests.

To investigate the effect of agonistic anti-CD137 antibodies on B-cell responses elicited by vaccination, we immunized mice with the model antigen chicken ovalbumin mixed in Alhydrogel, administered either subcutaneously (s.c.) or intraperitoneally (i.p.). Mice were treated with 100 μg of the agonistic anti-CD137 antibody (clone 3H3) or its isotype control on day 0 or day 4 post-immunization (Fig. 1A). Analysis of the spleens from i.p.-immunized mice and draining lymph nodes (dLNs) from s.c.-immunized mice at day 10 revealed a profound impairment of the antigen-specific B-cell response in the anti-CD137–treated animals. Total B-cell numbers remained comparable between both groups of mice and to those in unimmunized mice (Fig. EV1A), but germinal center (GC) B-cell percentages and absolute numbers were markedly reduced, as well as class-switched IgG1^+^ B cells (Figs. 1B,C and EV1B,C). This inhibitory effect was independent of the timing of antibody administration or the route of immunization and the secondary lymphoid organ (SLO) analyzed. We also observed a tendency toward defective plasmablast generation/differentiation in anti-CD137–treated mice immunized subcutaneously 10 days after immunization (Fig. 1D), observing a statistically significant difference when analyzed at later time-points (Figs. 1D and EV1D). Moreover, we analyzed the antibody titers generated against Ovalbumin in both groups of mice, observing a reduction in the anti-CD137 treated group of mice (Fig. 1E).

The abrogating effect on GC responses and IgG1 class-switch was also observed when mice were treated with a different agonistic anti-CD137 monoclonal antibody (1D8) binding to a different epitope on CD137 (Fig. EV1E).

**Figure 2:**
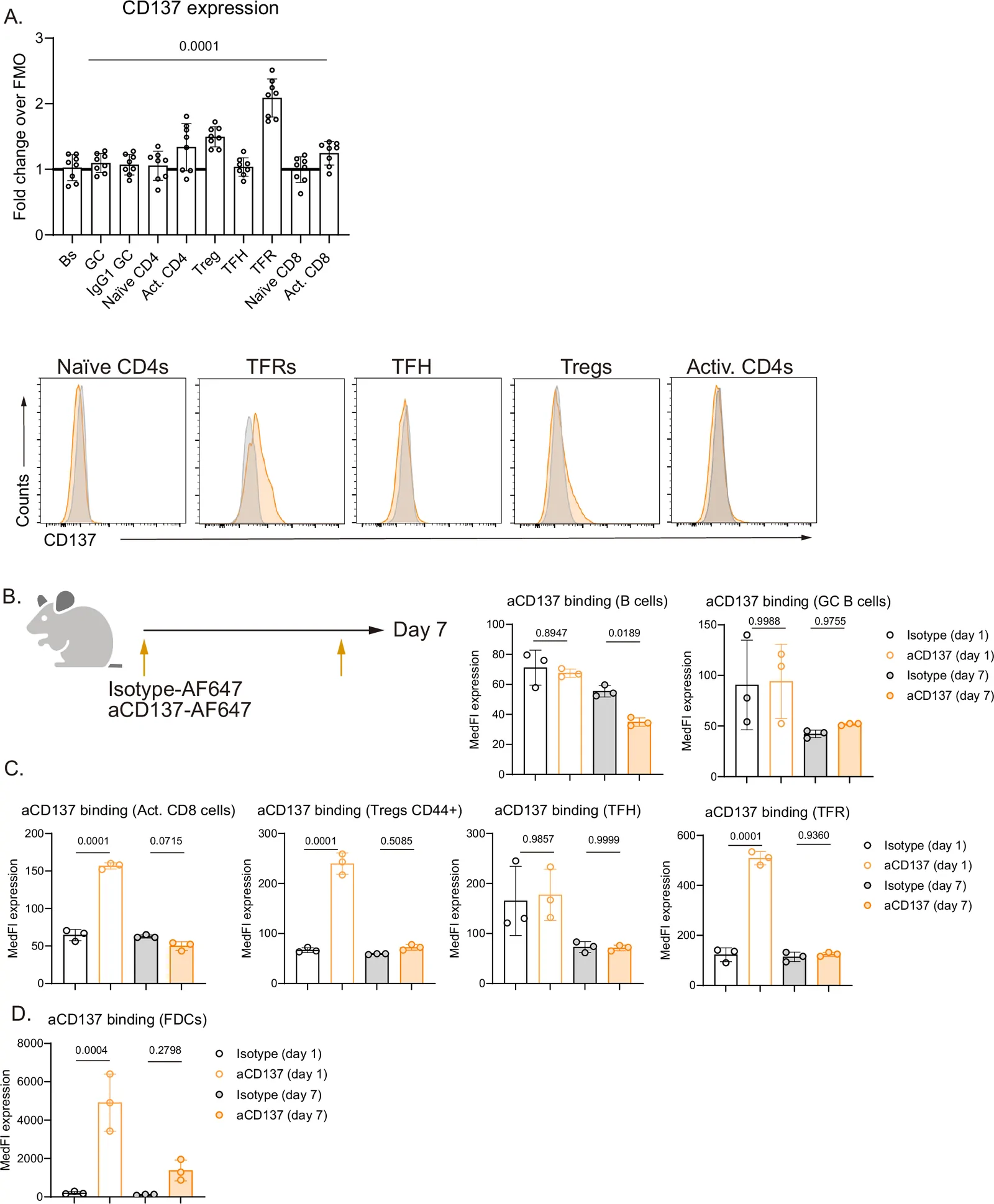
CD137 expression and cell binding distribution of the agonistic anti-CD137 antibody. (**A**) Quantification of CD137 expression (MFI) in mice immunized with ovalbumin in Alhydrogel. The graph shows the fold change in MFI relative to normalized FMO controls (dotted line). Representative histograms display anti-CD137 staining (orange) versus FMO (gray) across T-cell subsets (*n* = 8 mice/group; data pooled from 2 experiments). (**B**) Experimental workflow analyzing AF647-labeled anti-CD137 or isotype control antibody in vivo binding in immunized mice 24 h or 7 days post-injection. Quantification of antibody binding to total B cells and GC B cells (*n* = 3 mice/group; 1 independent experiment). (**C**) Quantification of anti-CD137 or isotype binding (MedFI) across T-cell subsets (*n* = 3 mice/group). (**D**) Quantification of antibody binding to FDCs (*n* = 3 mice/group). All quantitative data show means ± SD and were analyzed using one-way ANOVA with multiple comparisons. Source data are available online for this figure.

**Figure EV2:**
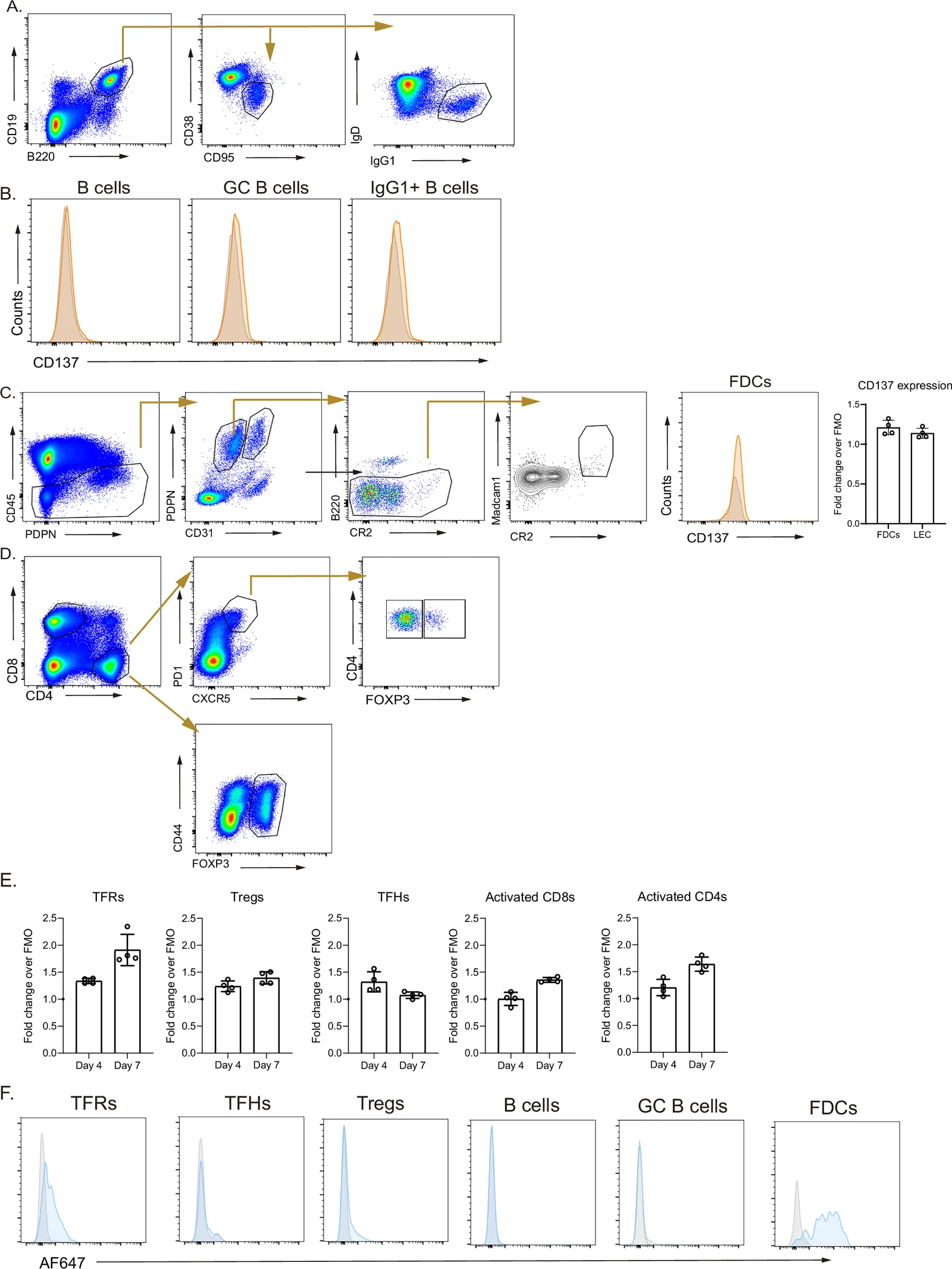
Analysis of CD137 expression and in vivo anti-CD137 targeting to lymphocyte populations and FDCs. (**A**) Flow cytometry strategy to analyze B cells, GC B cells and IgG1 CS B cells. (**B**) Histograms of CD137 expression (orange) and FMO (gray) on total B cells, GC and IgG1^+^ B cells. (**C**) Gating strategy to analyze FDCs. CD137 expression (orange) and FMO (gray) on FDCs. Quantification of CD137 expression (MFI) in mice immunized with ovalbumin in Alhydrogel. Graph shows the fold change in MFI relative to normalized FMO controls (*n* = 4 mice; 1 independent experiment). (**D**) Gating strategy to select Tfh (CXCR5 + PD-1 + ), Tfrs (CXCR5 + PD-1+ Foxp3+) and Tregs (CD4+ Foxp3+). (**E**) Quantification of CD137 expression (MFI) in mice immunized with ovalbumin in Alhydrogel for 4 and 7 days. Graph shows the fold change in MFI relative to normalized FMO controls (dotted line = 1) (*n* = 4 mice; 1 independent experiment). (**F**) Binding of AF647-labeled anti-CD137 (blue) and AF647-labeled IgG isotype (gray) to different dLNs immune populations. All quantitative data are shown as means ± SD.

To understand how anti-CD137 antibodies may impair humoral responses, we next analyzed the expression profile of CD137 in various stromal and lymphocyte populations involved in B-cell immunity (Figs. 2A and EV2A–D). CD137 expression was highest on regulatory T cells (Tregs) and follicular regulatory T cells (Tfr), a specialized subset that controls B-cell responses and shares features with Tregs. Interestingly, Tfr cells expressed even higher levels of CD137 than conventional Tregs or activated CD8⁺ T cells. Expression of CD137 on Tfrs, Tregs and activated lymphocytes was upregulated over time upon immunization with Ovalbumin (Fig. EV2E). To determine which cell types were targeted by the antibody in vivo, mice were injected with fluorescently labeled anti-CD137 or isotype control antibodies and analyzed their cellular distribution 24 h or 7 days later (Figs. 2B–D and EV2F). Flow cytometry revealed no binding of anti-CD137 to resting B cells or GC B cells (Fig. 2B), indicating a non-intrinsic mechanism of GC disruption. Instead, the antibody bound preferentially to activated CD8-T cells, Tregs, and Tfr cells, but not Tfh cells 24 h after injection (Fig. 2C,D). No detectable presence was found after 7 days as compared to the fluorescent isotype-injected group (Fig. 2C). Surprisingly, we also observed strong binding to follicular dendritic cells (FDCs) (Fig. 2D).

Thus, CD137 agonists profoundly impair the development of evolved B-cell responses without binding to GC B lymphocytes.

### A CD137 agonistic antibody interferes with FDC network expansion upon vaccination without altering antigen-display abilities of FDC

Because we observed specific binding of fluorescently labeled anti-CD137 to FDCs and previous reports have linked defective GC formation to FDC collapse (Hong et al, 2020), we next examined these cells in detail upon immunization and treatment with anti-CD137 mAb. FDCs form an entangled network within B-cell follicles that expands after immunization (Martinez-Riano et al, 2023; Pikor et al, 2020) and plays essential roles in B-cell survival, location, and activation by retaining and presenting antigen during the GC response (Allen and Cyster, 2008).

**Figure 3:**
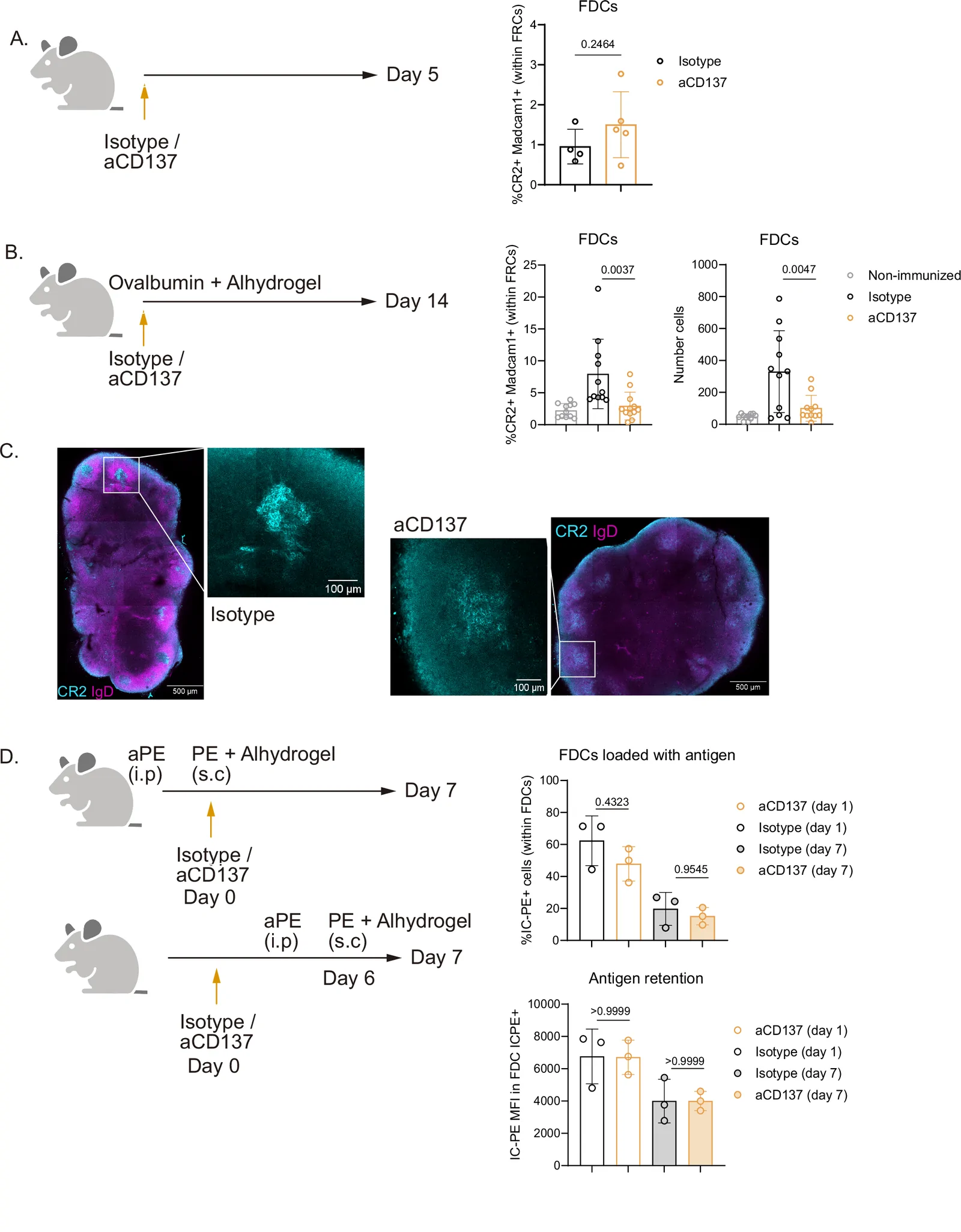
Anti-CD137 reduces FDC network expansion upon immunization but with preserved functionality. (**A**) Experimental workflow for analyzing FDCs in LNs from non-immunized mice treated i.p. with anti-CD137 (3H3) or its isotype control. Quantification of FDCs (CR2 + MAdCAM1+ within fibroblastic reticular cells -FRCs-) (*n* = 5 mice/group, 1 independent experiment). (**B**) Experimental workflow for analyzing FDCs in dLNs of mice immunized s.c. with ovalbumin and treated i.p. with anti-CD137 (3H3) or its isotype control. Quantification of FDCs (*n* = 11–12 mice/group; data pooled from 3 experiments). (**C**) Representative confocal images of clarified dLNs 14 days post-immunization showing FDC networks (anti-CR2, cyan) and B-cell follicles (anti-IgD, magenta). (**D**) Workflow for analyzing antigen display by FDCs at 24 h and 7 days after immunization with immune-complexed phycoerythrin (IC-PE). Mice were treated with anti-CD137 or isotype control on day 0. The upper graph shows the percentage of antigen-loaded FDCs (PE+) and the lower graph the quantity of antigen (MFI) (*n* = 3 mice/group, 1 independent experiment). All quantitative data show means ± SD and were analyzed using one-way ANOVA with multiple comparisons. Source data are available online for this figure.

**Figure EV3:**
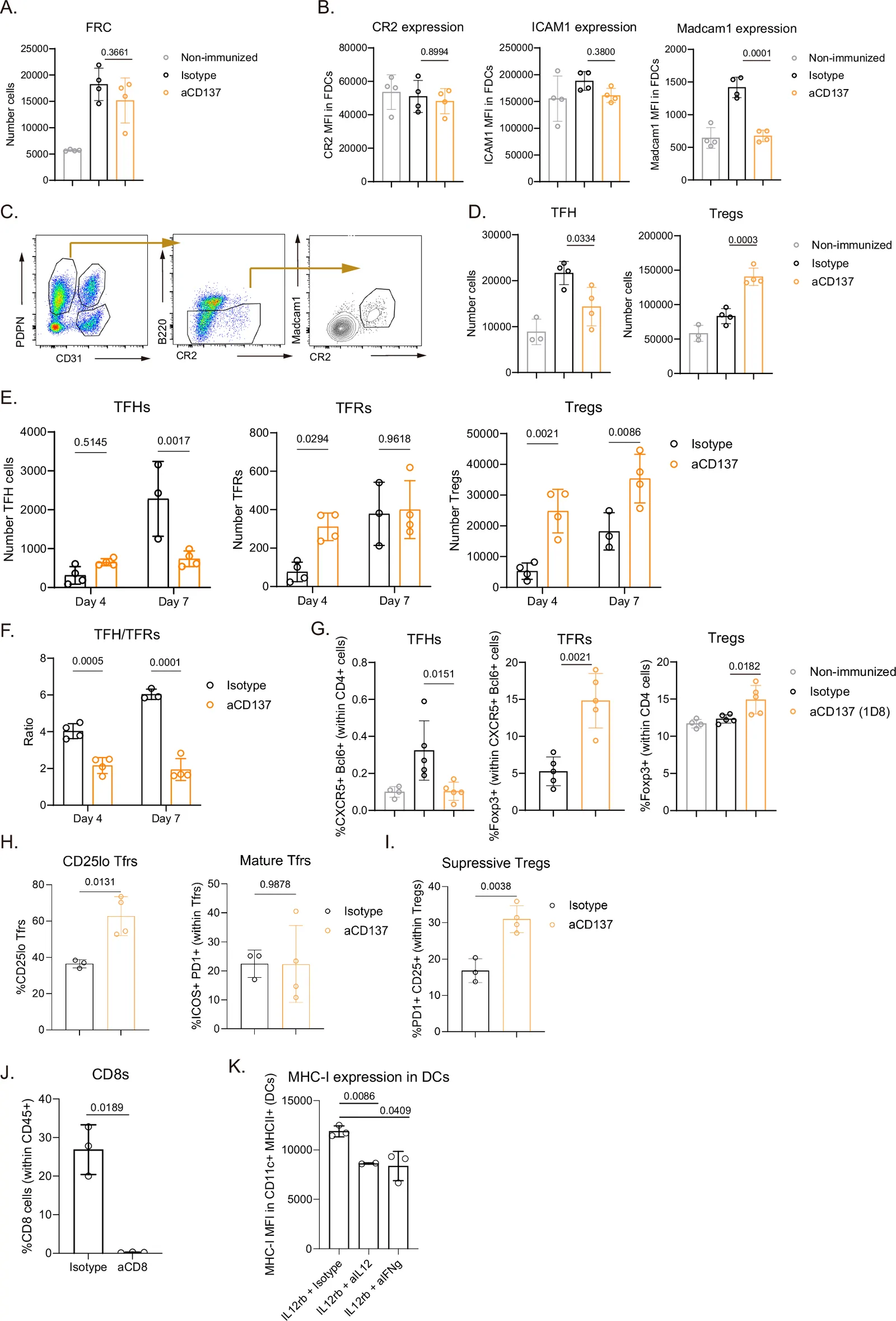
CD137-agonism in vivo modifies Treg and Tfh numbers in a manner independent of CD8-T cells, IFNγ and IL12. (**A**) Number of FRCs on the dLNs of non-immunized and immunized mice treated with anti-CD137 or its isotype-matched control. (**B**) Expression (MFI) of CR2, ICAM1, and MAdCAM1 on FDCs (*n* = 4 mice/group). (**C**) Gating strategy used to analyze FDCs on Fig. 3. (**D**) Number of Tfh and Treg cells on the dLNs of non-immunized and immunized mice treated with anti-CD137 or its isotype control (*n* = 4 mice/group). (**E**) Number of Tfhs, Tfrs and Tregs in immunized mice for 4 and 7 days treated with anti-CD137 or its isotype-matched control (*n* = 3–4 mice; 1 independent experiment). (**F**) Ratio Tfh/Tfrs at day 4 and 7 post-immunization (*n* = 4 mice). (**G**) Quantification of the percentage of Tfhs, Tfrs, and Tregs in non-immunized or immunized mice treated with anti-CD137 1D8 mAb or its isotype-matched control (*n* = 5 mice; 1 independent experiment). (**H**) Quantification of the percentage of Tfrs expressing low CD25 on the membrane (left graph) and ICOS + PD-1+ double-positive cells in mice immunized and treated with anti-CD137 or its isotype-matched control (*n* = 3–4 mice/group; 1 independent experiment). (**I**) Quantification of the percentage of PD-1 + CD25+ double-positive Tregs in mice treated as in (**H**). (**J**) Quantification of the percentage of CD8-T cells in the LNs of mice treated with the anti-CD8β depleting antibody or its isotype control (*n* = 3 mice/group; 1 independent experiment). (**K**) Quantification of the MFI of MHC-I expressed by DCs in mice injected with recombinant IL-12 (10 µg) and treated with neutralizing anti-IL12, anti-IFNγ or isotype control at days -1 and 1 (*n* = 2–3 mice/group; 1 independent experiment). All quantitative data are shown as mean ± SD and analyzed using one-way ANOVA with multiple comparisons or Welch’s *t*-test.

Non-immunized mice were treated with a single dose of anti-CD137 or isotype control. Five days later we analyzed the FDCs, observing no differences in their percentage when comparing both groups of mice (Fig. 3A). However, when mice were immunized subcutaneously with ovalbumin in Alhydrogel and treated either with a single dose of anti-CD137 or isotype control antibody (Figs. 3B and EV3C), we observed that FDC numbers increased upon immunization in isotype-treated mice, but this expansion was completely absent in anti-CD137 treated animals. This group displayed FDC numbers similar to those of naïve mice (Fig. 3B). In contrast, the number of fibroblastic reticular cells (FRCs) remained unchanged (Fig. EV3A). FDCs from anti-CD137-treated mice also exhibited a less mature phenotype, with reduced ICAM1 and MAdCAM1 expression as compared to those in isotype-treated controls (Fig. EV3B), although anti-CD137-treated mice were still able to form follicular FDC networks (Fig. 3C).

Because GC B-cell maintenance depends on continuous antigen acquisition and presentation from FDCs, we next asked whether anti-CD137 treatment affected the ability of FDCs to display antigen. Mice were immunized with fluorescently labeled antigen (phycoerythrin, PE) in immunecomplex form and analyzed 24 h and 7 days later (Fig. 3D). The proportion of antigen-loaded FDCs and the amount of retained antigen were comparable between anti-CD137 and isotype-treated groups (Fig. 3D).

Together, these results indicate that although agonistic anti-CD137 treatment prevents the immunization-induced expansion and maturation of the FDC network, the agonistic antibody does not change the percentages of viable FDCs at steady-state, neither their functional role capturing and presenting antigens to B cells.

### CD137 agonists induce expansions of Tregs and Tfrs populations

**Figure 4:**
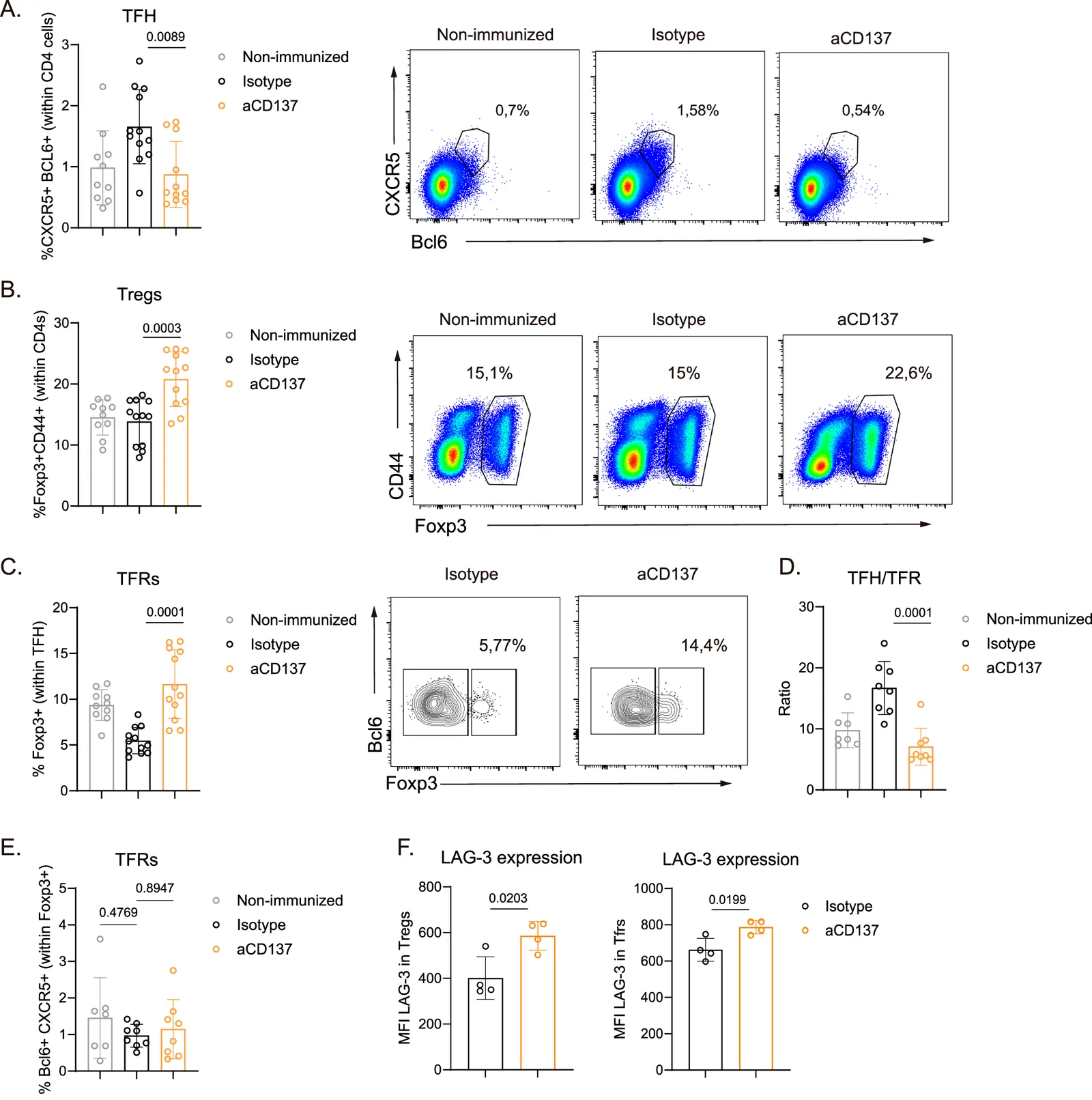
Anti-CD137 induces expansion of Treg and Tfr populations and disrupts the Tfh/Tfr balance. (**A**) Quantification and flow cytometry panels of the percentage of Tfh cells (Bcl6 + CXCR5+) in non-immunized or immunized mice treated with anti-CD137 or its isotype control (*n* = 9–12 mice/group, data pooled from 3 experiments). (**B**) Quantification and flow cytometry panels of the percentage of Treg cells (Foxp3+) in non-immunized or immunized mice treated with anti-CD137 or its isotype control (*n* = 10–12 mice/group, data pooled from 3 experiments). (**C**) Quantification and flow cytometry panels of the percentage of Tfr cells (Foxp3+ within TFHs) in non-immunized or immunized mice treated with anti-CD137 or its isotype control (*n* = 10–12 mice/group, data pooled from 3 experiments). (**D**) Ratio of Tfh/Tfrs (*n* = 7–-8 mice/group, data pooled from 2 experiments). (**E**) Quantification of the percentage of Tfr cells (Bcl6 + CXCR5+) within Foxp3+ cells (*n* = 7–8 mice/group, data pooled from 2 experiments). (**F**) LAG-3 expression on Tregs (left graph) or Tfrs (right graph) (*n* = 4/group mice; 1 independent experiment). All quantitative data show means ± SD and were analyzed using one-way ANOVA with multiple comparisons or *t*-tests. Source data are available online for this figure.

Given the changes observed in B-cell responses after anti-CD137 treatment, we examined whether differentiation of T follicular helper (Tfh) cells was affected. Fourteen days post-immunization, anti-CD137–treated mice displayed Tfh frequencies comparable to those of non-immunized controls and approximately twofold decrease as compared with isotype-treated mice (Figs. 4A and EV3D). Consistent with the observed antibody binding to Tregs and Tfr cells, we analyzed these T lymphocyte subsets in greater detail. Tregs and Tfr cells are known to exert immunosuppressive effects through inhibitory co-receptors such as LAG-3 and CTLA-4, among different mechanisms (Andrews et al, 2017; Cavaco et al, 2025). Tfr cells, characterized by Bcl6-dependent upregulation of CXCR5, can enter B-cell follicles and directly suppress local B-cell responses. We found that anti-CD137 treatment increased both the frequency and absolute numbers of Tregs (Figs. 4B and EV3D) and the frequency of Tfr cells (Fig. 4C), leading to an imbalanced Tfh/Tfr ratio, a hallmark of defective GC responses (Fig. 4D) (Sage et al, 2014). This increase in Tfr cells was not due to an enhanced differentiation rate from Treg precursors (Fig. 4E). Differences in Tfr and Treg cell numbers, as well as in the Tfh/Tfr ratio, were already evident by day 4 after treatment and, in the case of Tregs, remained apparent 7 days later (Fig. EV3E,F).

Importantly, the effect of anti-CD137 3H3 agonistic antibody on TFHs, Tfrs and Tregs was also observed using a second different monoclonal antibody (1D8) (Fig. EV3G). Furthermore, Tregs and Tfrs from anti-CD137–treated mice expressed higher levels of LAG-3 on their surfaces (Fig. 4F). Enhanced B-cell suppressive potential was also supported by the increased proportion of CD25low-expressing Tfr cells in the anti-CD137-treated mice, which correspond to GC-localized Tfr cells (Fig. EV3H) (Wing et al, 2017). Likewise, the frequency of activated immunosuppressive Tregs (PD-1 + CD25 + ) was increased in the anti-CD137-treated group (Fig. EV3I). However, we did not detect differences in the proportion of ICOS + PD-1+ Tfr cells, a population proposed to represent a more mature Tfr subset, at least in humans (Fig. EV3H) (Ribeiro et al, 2025).

### Anti-CD137-mediated GC inhibition is independent of CD8-T cells, IL-12 and IFN-γ

**Figure 5:**
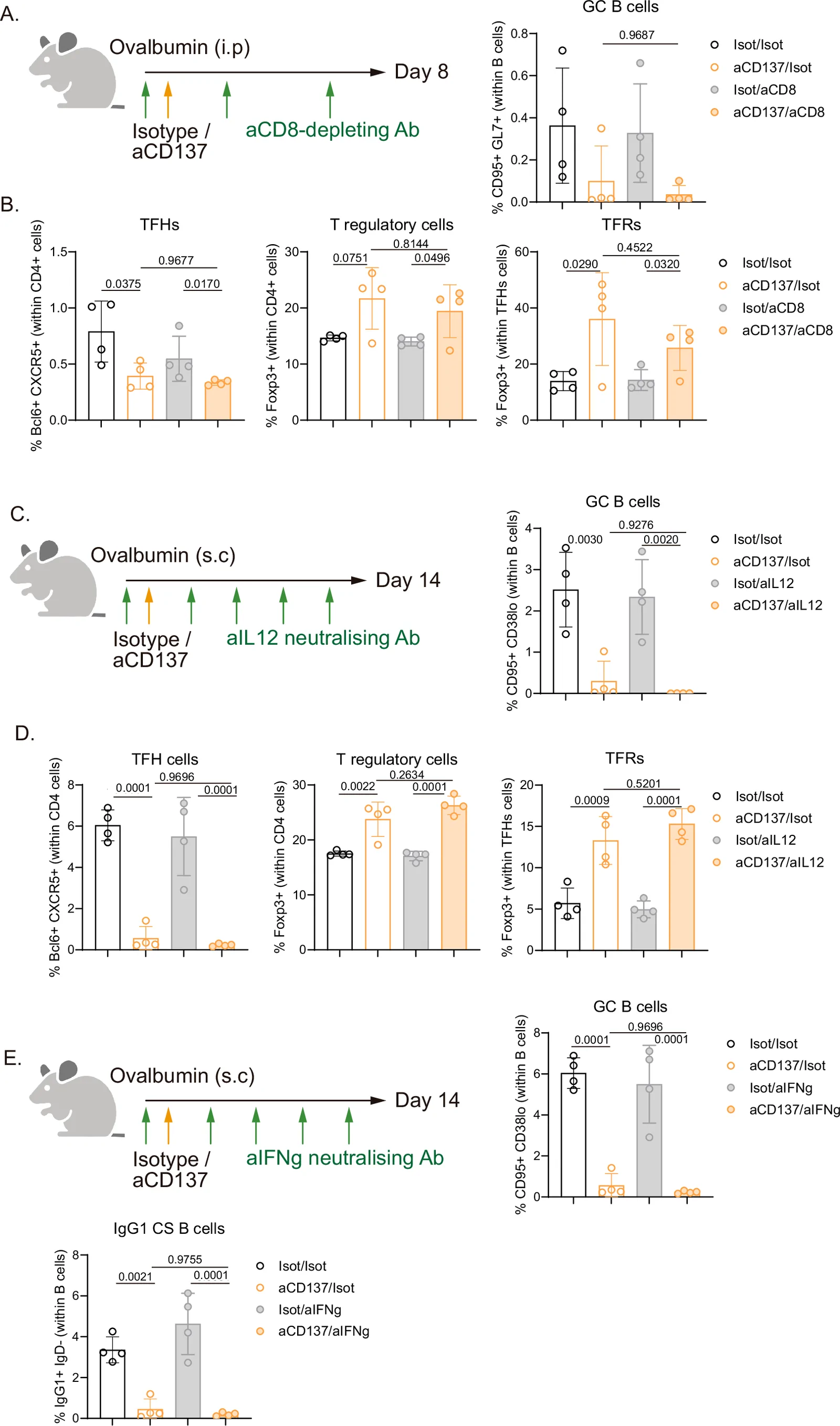
Anti-CD137-dependent GC inhibition is independent of CD8 + T cells, IL-12, and IFNγ. (**A**) Experimental workflow. Mice were immunized i.p with ovalbumin and injected with 100 µg of anti-CD8β depleting antibody (isotype-treated in gray; anti-CD137-treated in filled orange) or its isotype control (isotype-treated in black; anti-CD137-treated in unfilled orange) at days -1, 2, and 5. The graph shows the quantification of the GC B cells in the four groups of mice (*n* = 4 mice/group; 1 independent experiment). (**B**) Quantification of the percentage of Tfh, Tregs and Tfrs (*n* = 4 mice/group). (**C**) Experimental workflow. Mice were immunized s.c with ovalbumin and injected with anti-IL12p40 neutralizing antibody (isotype-treated in gray; anti-CD137-treated in filled orange) or its isotype-matched control (isotype-treated in black; anti-CD137-treated in unfilled orange) every 2–3 days starting the day before immunization. The graph shows the quantification of the GC B cells in the four groups of mice (*n* = 4 mice/group; 1 independent experiment). (**D**) Quantification of the percentage of Tfh, Tregs and Tfrs in IL-12 neutralized mice (*n* = 4 mice/group; 1 independent experiment). (**E**) Experimental workflow. Mice were immunized s.c with Ovalbumin and injected with anti-IFNγ neutralizing antibody (isotype-treated in gray; anti-CD137-treated in filled orange) or its isotype-matched control (isotype-treated in black; anti-CD137-treated in unfilled orange) every 2–3 days starting the day before immunization. Quantification of the percentage of GC B cells and IgG1^+^ class-switched B cells (*n* = 4 mice/group; 1 independent experiment). All quantitative data show means ± SD and were analyzed using one-way ANOVA with multiple comparisons. Source data are available online for this figure.

CD137 agonists are known to enhance cytotoxic T lymphocytes (CTL) and Th1-type immune responses, which in turn may modify B-cell responses through different mechanisms. To test whether CD8^+^ T cells contribute to the GC disruption observed upon anti-CD137 treatment, we injected immunized mice with a depleting anti-CD8β antibody or isotype control, together with anti-CD137 or its isotype control (Fig. 5A). The anti-CD8β antibody dosing regimen achieved near-complete depletion of CD8-T cells in secondary lymphoid organs (Fig. EV3J). Eight days post-immunization, the spleens of the mice were analyzed, showing that anti-CD137-treated mice had a severely reduced GC response (Fig. 5A), reduced Tfh frequencies, and increased Tregs and Tfrs (Fig. 5B), consistent with previous results. However, CD8 depletion did not rescue the GC defect or alter the expansion of Tregs and Tfrs, indicating that the defect in GC formation upon anti-CD137 treatment is not dependent on CD8 + T cells.

CD137 ligation on dendritic cells (DCs) by activated T cells promotes DC activation and IL-12 secretion, which can drive type I immune responses. As IL-12 has been reported to suppress Tfh differentiation (Elsner and Shlomchik, 2019; Elsner et al, 2024), we next tested whether IL-12 contributed to the GC blockade by CD137 agonists. First, we assessed the in vivo efficacy of a neutralizing anti–IL-12 p40 antibody. Mice were administered recombinant IL-12 alone or together with the neutralizing antibody, and MHC-I expression on DCs was used as a functional readout of IL-12 activity. This approach confirmed the neutralizing capacity of the antibody used (Fig. EV3K). Mice were immunized and treated with anti-CD137, as previously done, and received neutralizing anti–IL-12 p40 or isotype control antibodies for two weeks (Fig. 5C). Regardless of IL-12 neutralization, anti-CD137–treated mice kept exhibiting defective GC responses, with evidence for impaired Tfh differentiation, and increased Treg and Tfr frequencies (Fig. 5D). Thus, IL-12 is not responsible for the GC inhibition induced by CD137 stimulation with agonist antibodies.

Because one of the main consequences of CD137 signaling is an increase in IFNγ secretion by CD8⁺ T and NK cells, we next examined whether this cytokine could influence GC formation. Mice were immunized, treated with anti-CD137, and injected with a neutralizing anti-IFNγ antibody, whose neutralizing ability was also tested (Fig. EV3K). GC responses remained abrogated in anti-CD137-treated mice independently of IFNγ blockade (Fig. 5E).

Collectively, these results demonstrate that anti-CD137-induced GC disruption is independent of CD8^+^ T cells and the pro-inflammatory cytokines IL-12 and IFN-γ, two of the major hallmarks of CD137 co-stimulation.

### Anti-CD137-dependent GC inhibition depends on Foxp3+ Tregs and Tfrs

**Figure EV4:**
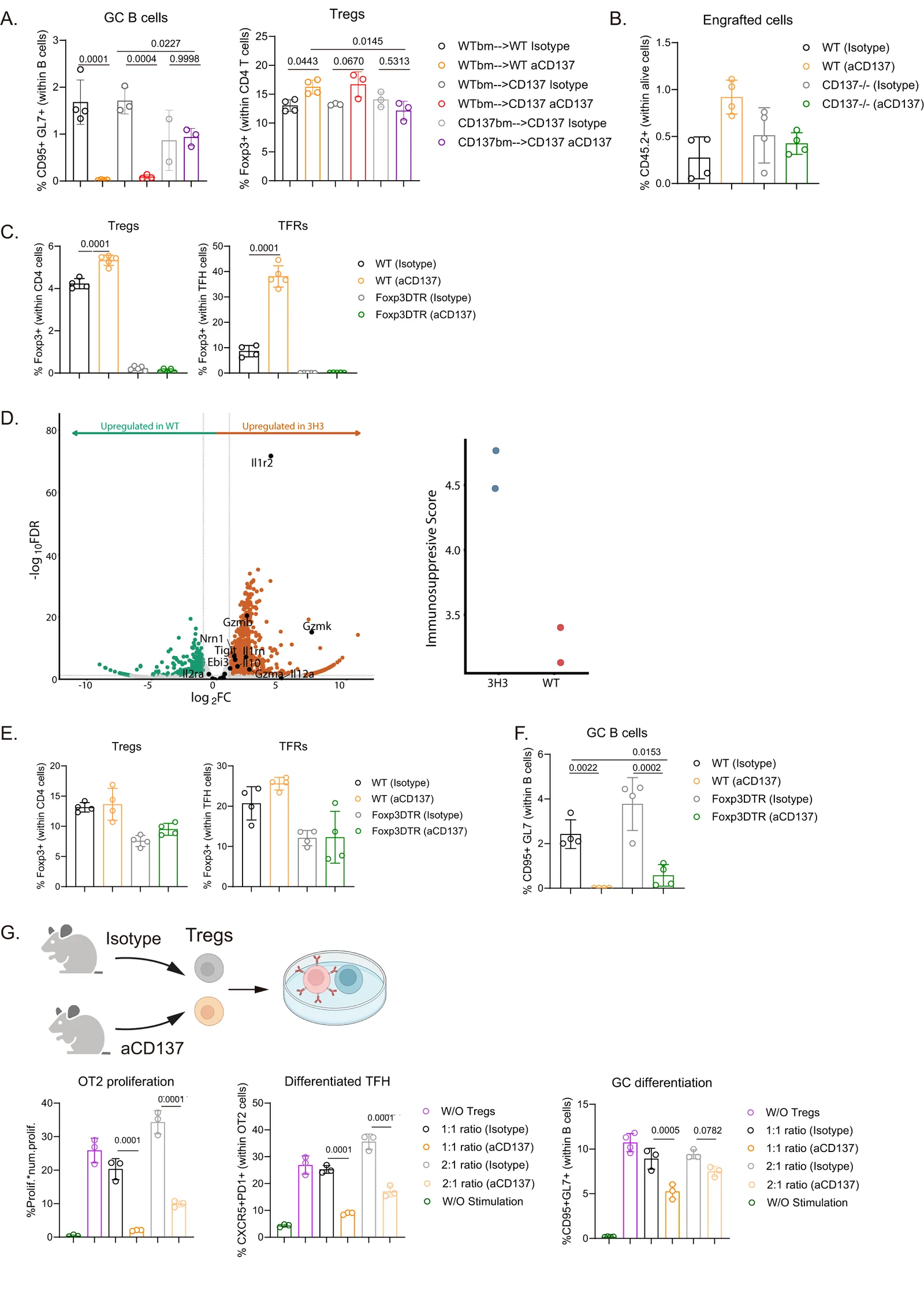
CD137-agonism acting in bone marrow-derived cells increases Treg/Tfr immunosuppressive activity. (**A**) Percentage of GC B cells and Tregs in the indicated bone marrow reconstituted mice (*n* = 2–4 mice/group; 1 independent experiment). (**B**) Percentage of engrafted CD45.2+ cells on CD45.1+ host mice 14 days after immunization (*n* = 4 mice/group; 1 independent experiment). (**C**) Percentage of Tregs and Tfrs on the LNs of WT and Foxp3-DTR mice injected with anti-CD137 or its isotype and 660 ng of DTx (*n* = 4 mice/group; representative of two independent experiments). (**D**) Volcano plot showing the magnitude of the transcriptional changes (log2 fold change) together with the statistical significance (−log10 FDR) of the genes expressed by Tregs from isotype- or anti-CD137-treated mice. In black, highlight those genes that participate in the immunosuppressive gene score. The right graph shows the immunosuppressive score of the sequenced Foxp3+ cells. (**E**) Percentage of Tregs and Tfrs on the LNs of WT and Foxp3-DTR mice injected with anti-CD137 or its isotype and 66 ng of DTx (*n* = 4 mice/group; one independent experiment). (**F**) WT and Foxp3-DTR mice treated as in (**C**) but with 10× lower DTx dose (66 ng/dose) (*n* = 4 mice/group). (**G**) Co-culture assay workflow. Foxp3-GFP mice were injected with anti-CD137 or isotype control; 4 days later, GFP⁺ cells were sorted and co-cultured for 3 days with purified B cells, CTV-labeled OT-II CD4 T cells, and 1 μm beads coated with anti-IgM F(ab’)_2_ and ovalbumin. Graphs show OT-II proliferation, as well as Tfh (CXCR5 + PD-1 + ) and GC B cells (CD95 + GL7 + ) percentages. Tregs were added at CD4 T:Treg ratios of 1:1 or 2:1; cultures without Tregs (magenta) and without bead stimulation (green) served as controls (representative of two independent experiments). All quantitative data were shown as means ± SD and analyzed using one-way ANOVA with multiple comparisons.

To in-depth elucidate the mechanism by which anti-CD137 treatment alters B-cell responses, we tested whether hematopoietic or radioresistant non-hematopoietic cells were responsible. WT and CD137−/− mice were lethally irradiated and reconstituted with bone marrow from WT or CD137−/− donors (Fig. EV4A). Two months later, mice were immunized and treated with anti-CD137 or isotype control as previously done. Ten days after immunization, lymph nodes were analyzed. We observed that GC responses were impaired, and Treg frequencies increased in mice reconstituted with WT bone marrow, regardless of the recipient genotype. In contrast, these effects were absent in the chimeras receiving CD137−/− bone marrow (Fig. EV4A), indicating that the anti-CD137 effect was exerted on hemopoietic-lineage cells.

**Figure 6:**
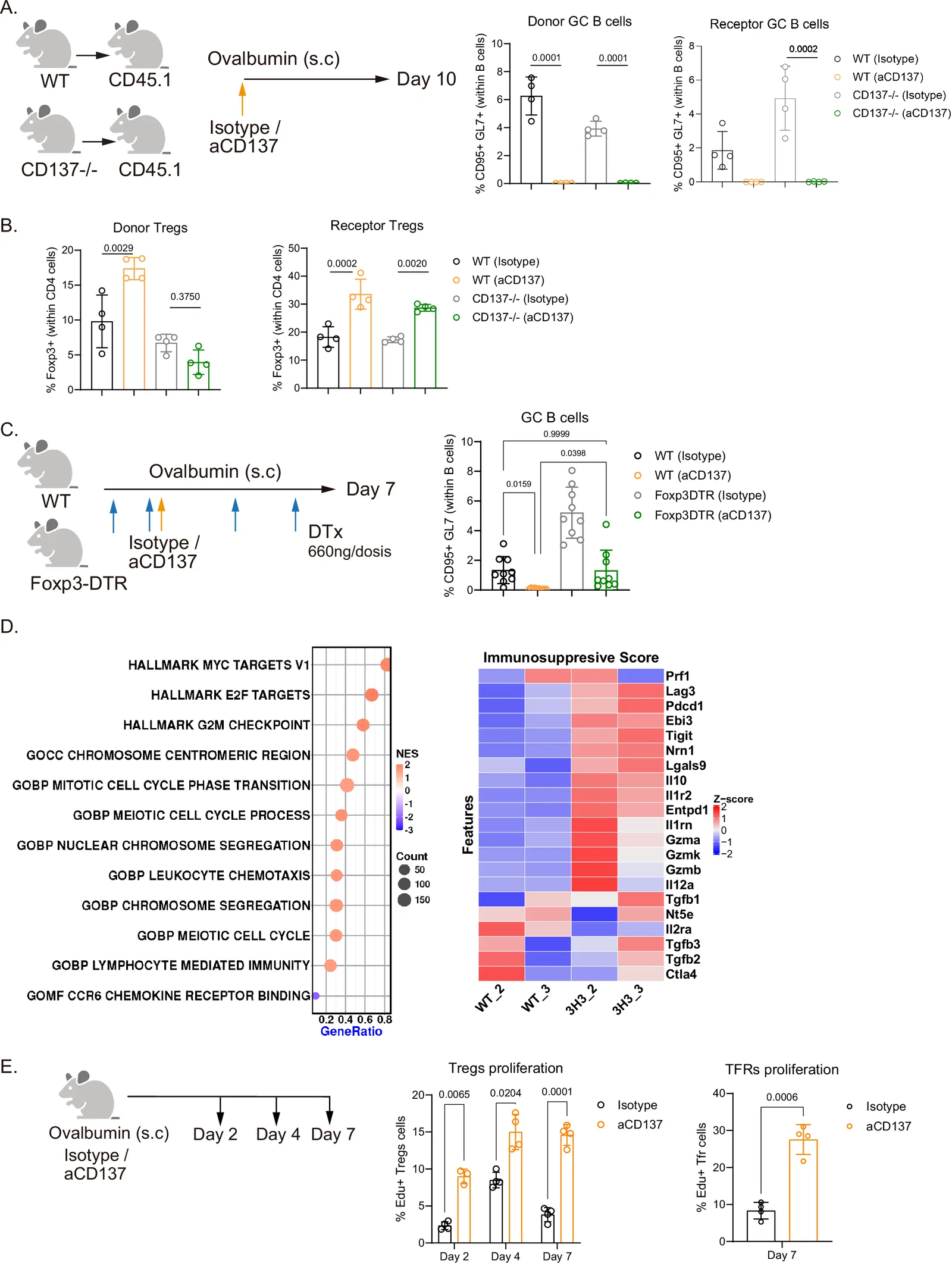
CD137-dependent GC inhibition is partially dependent on Foxp3+ T cells. (**A**) Workflow for adoptive transfer experiments. Splenocytes from WT or CD137−/− mice were transferred i.v. into congenic CD45.1 recipients, which were immunized s.c. with ovalbumin and treated i.p. with anti-CD137 or isotype control. Ten days later, dLNs were analyzed. Graphs show GC B-cell quantification in donor WT (isotype in black; anti-CD137 in gray) or CD137−/− (isotype in orange; anti-CD137 in green) and in recipient CD45.1 cells (*n* = 4 mice/group, 1 independent experiment). (**B**) Quantification of Tregs from donor WT or CD137−/− mice (left) and recipient CD45.1 cells (right) (*n* = 4 mice/group). (**C**) WT and Foxp3-DTR mice were treated with diphtheria toxin (DTx; 660 ng/dose i.p. every 2–3 days), immunized s.c. with ovalbumin, and treated with anti-CD137 or isotype control. Quantification of GC B cells and IgG1^+^ class-switched B cells (n = 9-10 mice/group; data pulled from 2 experiments). (**D**) GSEA plot (left panel) and heat-map (right panel) showing the relative expression of the immunosuppressive signature genes from RNA-seq analysis of Foxp3+ cells (Foxp3-GFP+ hCD2-RFP+ cells) sorted from isotype- or anti-CD137-treated mice for 4 days prior to sacrifice (*n* = 2 mice/sample; 2 samples/treatment). (**E**) Workflow for in vivo Edu-based proliferation assay. Mice immunized with Ovalbumin were treated with anti-CD137 or isotype control for 2, 4, or 7 days. Quantification shows the Edu incorporation at 2, 4, and 7 days on Tregs (left graph) and at 7 days on Tfrs (right graph) (*n* = 4 mice/group). All quantitative data show means ± SD and analyzed by one-way ANOVA and Krustal–Wallis tests with multiple comparisons or *t*-tests. Source data are available online for this figure.

To further dissect this mechanism, we performed adoptive transfer experiments, injecting WT or CD137−/− CD45.2^+^ splenocytes into CD45.1^+^ recipient mice that were then immunized and treated with anti-CD137 (Figs. 6A and EV4B). GC impairment occurred in anti-CD137-treated mice regardless of whether donor B cells expressed or not CD137, further indicating that the effect is not mediated by direct antibody binding to B cells. However, when Treg cells lacked CD137, their expansion was not observed, in contrast to WT donor or host cells (Fig. 6B), strongly suggesting that the antibody directly promotes Treg expansion via CD137-agonism engagement.

Because Tregs, and particularly Tfrs, can suppress humoral responses through multiple mechanisms, we next tested whether their expansion contributes to the GC defect observed. We used Foxp3-DTR mice, which express the diphtheria toxin receptor under Foxp3 promoter control, allowing selective depletion of Foxp3⁺ regulatory cells upon diphtheria toxin (DTx) administration (Fig. 6C). WT and Foxp3-DTR mice were treated with DTx every two days, immunized with ovalbumin, and treated with anti-CD137 or its isotype control. After 7 days, dLNs showed as expected a complete depletion of Tregs and Tfrs (Fig. EV4C) and, importantly, a partial restoration of GC B-cell responses in the anti-CD137-treated mice. GC-reaction parameter reached levels similar to those of WT isotype controls, though still lower than Foxp3-DTR isotype controls. This indicates that GC inhibition by anti-CD137 is at least partially dependent on Tregs/Tfrs.

To explore further the possible effects on Tregs induced by the anti-CD137 agonism, Foxp3-GFP hCD2-RFP mice were injected with isotype or anti-CD137 agonist mAb. Four days later, Foxp3-expressing cells from both groups of mice were sorted and analyzed transcriptomically. GSEA shows that anti-CD137-treated Foxp3+ cells have an enhanced expression of genes regulating proliferation and cell cycle (Fig. 6D), supporting the observation of increased numbers of Tregs and Tfrs found after treatment (Figs. 4B,C and EV3D). To determine whether anti-CD137 treatment directly enhances Treg and Tfr proliferation, mice were immunized with ovalbumin as described above and treated with either anti-CD137 or an isotype control for different durations. EdU incorporation was assessed at days 2, 4, and 7 post-immunization (Fig. 6E). Increased proliferation was observed in both Treg and Tfr populations, supporting the transcriptomic findings. To explore further differences that might affect the humoral response, we set up an immunosuppressive gene score based on Treg and Tfrs literature (Cavaco et al, 2025; Dikiy and Rudensky, 2023; Ding et al, 2019; Gonzalez-Figueroa et al, 2021; Maceiras et al, 2017; Wing et al, 2017; Ye et al, 2021). Anti-CD137-treated Foxp3-expressing cells show an increased immunosuppressive score (Figs. 6D and EV4D), with the upregulation of genes such as Il10, Il1r2, Granzymes and Lag3, also observed at the protein level (Fig. 4F), in agreement with what we had previously observed (Fig. EV3H,I).

To distinguish between effects due to Treg expansion versus enhanced suppressive activity, Foxp3-DTR mice were treated with a ten-fold lower DTx dose than the one previously used to only partially reduce, but not fully deplete, Tregs/Tfrs. This strategy achieved ~50% reduction as compared to WT mice (Fig. EV4E). Despite reduced Foxp3+ numbers, anti-CD137-treated mice still displayed a four-fold decrease in GC B cells compared with respect to untreated WT animals, indicating that even in reduced numbers, CD137-agonism was enhancing the Foxp3-mediated immunosuppressive activity (Fig. EV4F).

To further validate the RNA-seq data and assess whether Foxp3-expressing cells from anti-CD137-treated mice were functionally more suppressive, Foxp3-GFP hCD2-RFP mice were treated with anti-CD137 or isotype control, and Foxp3-expressing cells were sorted four days later and co-cultured with purified B cells stimulated with F(ab’)_2_ anti-IgM and ovalbumin-coated microbeads and OT-II CD4⁺ T cells labeled with CTV to assess their proliferation levels (Fig. EV4G). This co-culture system mimics key GC events, including B-cell antigen uptake, presentation to T cells, and reciprocal differentiation into GC B and Tfh cells. After 3 days of co-culture with different Foxp3:T cell ratios, co-cultures containing Tregs from anti-CD137–treated mice more efficiently suppressed T-cell proliferation and Tfh/GC differentiation as compared to controls, indicating an enhanced immunosuppressive functional activity on a per-cell basis.

According to our experimentation, agonistic anti-CD137 antibodies promote Treg/Tfr expansion and an increase in their suppressive performance on B-cell responses. These mechanisms collectively contribute to GC disruption.

### Agonist anti-CD137 antibodies disrupt B-cell responses associated with tumor-associated tertiary lymphoid structures and prevent anti-drug antibody formation

**Figure 7:**
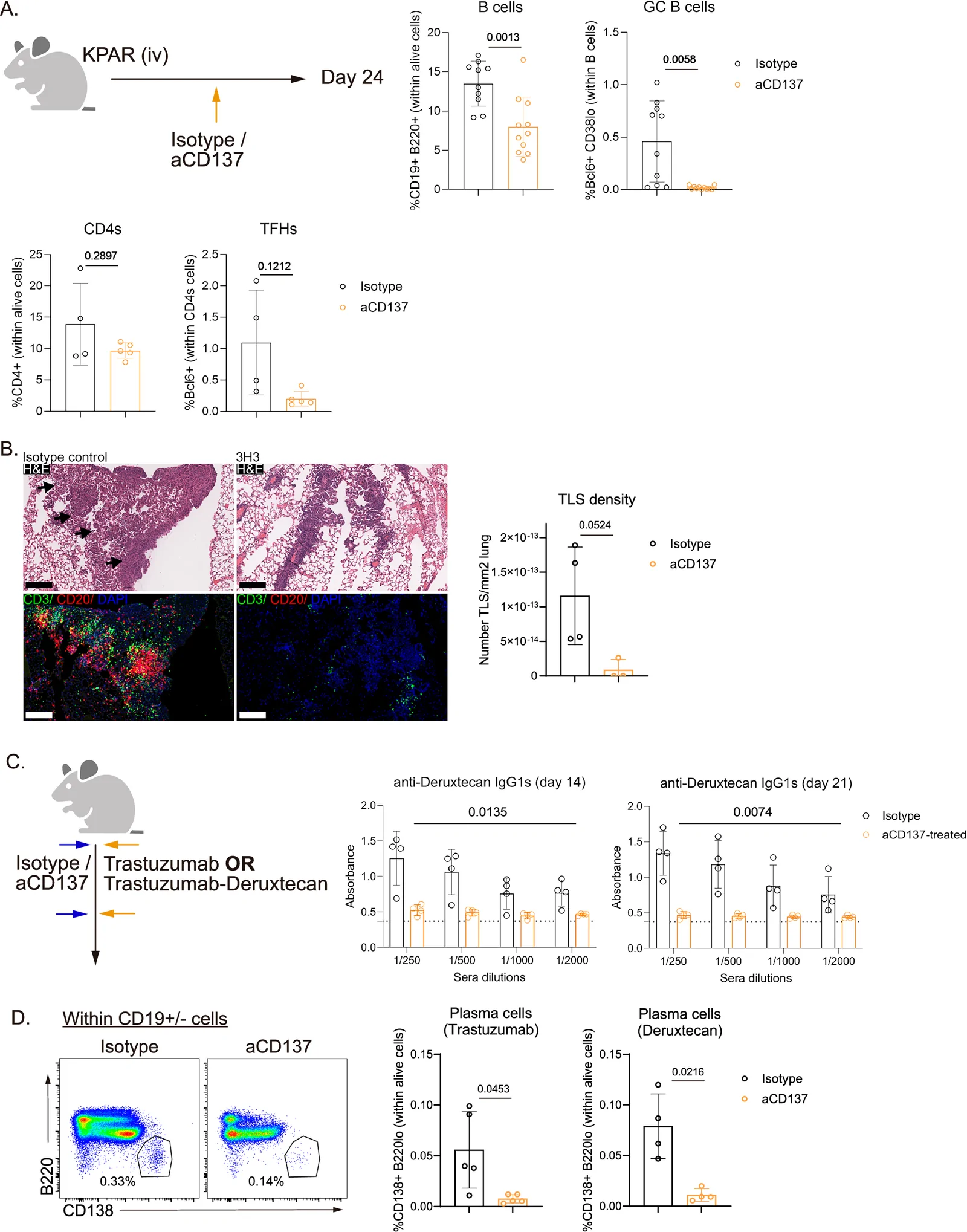
Anti-CD137 disrupts tumor-associated TLS and ADA-associated humoral responses to therapeutic antibodies. (**A**) Mice were injected i.v with 300.000 KPAR cells and two weeks later treated with anti-CD137 or its isotype control. Quantification of lung B cells, GC B cells (*n* = 10 mice/group; data pulled from 2 experiments), CD4 and Tfh (*n* = 4 mice/group) was performed at day 24. (**B**) Quantification of the density of TLS (number TLS/mm^2^ lung tissue) and representative images showing the tumoral areas (H&E) and the B-cell and T-cell immune infiltrate (IF; anti-CD20 is shown in red; anti-CD3 is shown in green; and DAPI is shown in blue). Arrows point at TLS location within the lung/tumor area (*n* = 3–4 mice/group). (**C**) Experimental workflow for the analysis of ADAs. Mice were immunized with Trastuzumab or Trastuzumab-Deruxtecan subcutaneously and treated as indicated with anti-CD137 or its isotype-matched control. Two weeks later, mice were reimmunized. Sera from the mice were obtained at day 14, before reimmunization, and 21 post-reimmunization and analyzed by ELISA for anti-Trastuzumab IgG1 antibodies. The dotted line shows the background level in non-immunized mice (*n* = 4 mice/group; 1 independent experiment). (**D**) Bone marrow cells from (**C**) were analyzed for the presence of plasma cells (CD138^+^ B220low) (*n* = 4–5 mice/group; 1 independent experiment). All quantitative data were shown as means ± SD and analyzed using two-way ANOVA  or *t*-tests. Source data are available online for this figure.

**Figure EV5:**
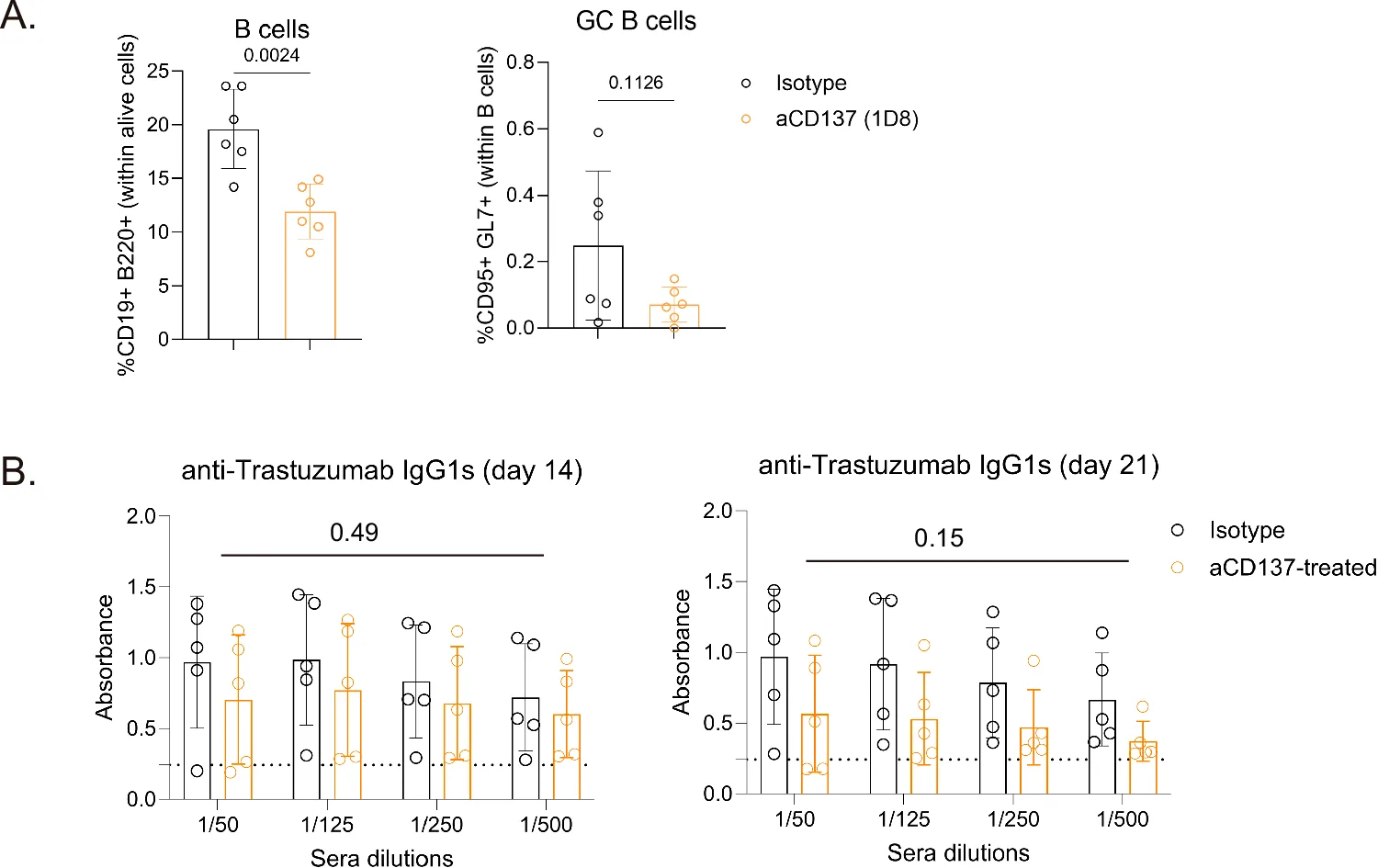
Anti-CD137 disrupts tumor-associated TLS and ADA-associated humoral responses. (**A**) Quantification of lung B cells and GC B cells in mice bearing KPAR tumors treated with isotype or anti-CD137 1D8 mAb (*n* = 5–6 mice/group; 1 independent experiment). (**B**) Absorbance levels of IgG1 anti-Trastuzumab antibodies in the sera of mice immunized with Trastuzumab and treated with isotype or anti-CD137 antibody after 14 days or 21 days post-immunization. The dotted line shows the background level of antibodies from non-immunized mice (*n* = 5 mice/group; 1 independent experiment). Quantitative data were shown as means ± SD and analyzed using two-way ANOVA or *t*-tests.

While humoral responses primarily occur in SLOs, chronic inflammatory conditions such as cancer or autoimmunity, can induce ectopic lymphoid aggregates known as tertiary lymphoid structures (TLSs) located outside lymphoid organs. TLSs consist of a B-cell-rich area adjacent to T-cell-rich zones (Schumacher and Thommen, 2022). Mature TLSs develop GC-like structures that actually closely resemble those in SLOs. In cancer, TLS presence correlates with improved survival and response to immune checkpoint blockade (Cabrita et al, 2020; Fridman et al, 2022; Helmink et al, 2020; Petitprez et al, 2020). To assess the effect of anti-CD137 therapy on tumor-associated TLS-dependent B-cell responses, we used the non-small cell lung carcinoma (NSCLC) KPAR cell line, which derives from the KP line, but has been reported to be more immunogenic (Boumelha et al, 2022). Two weeks after orthotopic (i.v) tumor inoculation, mice were treated with anti-CD137 or isotype control (Fig. 7A). Flow cytometry analysis of their excised lungs revealed that anti-CD137-treated mice contained fewer infiltrating B cells and reduced frequencies of GC B cells (Fig. 7A), with a trend for a concomitant decrease in Tfh cell content. Multiplex immunofluorescence microscopy confirmed the absence of TLS organization in the lungs of mice two weeks after anti-CD137 treatment (Fig. 7B). Reduction of TLS-associated B-cell response was also observed using a different anti-CD137 agonistic mAb (1D8) (Fig. EV5A).

Multiple standard-of-care cancer treatments rely on recombinant antibodies, now including multiple formats of bispecifics and antibody-drug conjugates (ADCs). With this notion in mind, we investigated whether anti-CD137 antibodies could also modulate B-cell responses elicited against therapeutic recombinant immunoglobulins, including popular ADCs, which often induce anti-drug antibodies (ADAs) that compromise therapeutic efficacy. As a proof-of-concept, we used trastuzumab, an anti-HER2 antibody, and trastuzumab-deruxtecan, an ADC in which trastuzumab is conjugated to the topoisomerase I inhibitor deruxtecan. Mice were immunized with trastuzumab or trastuzumab-deruxtecan and treated with anti-CD137 or isotype control on days 0, and reimmunized at day 14 (Fig. 7C). Blood samples were drawn at day 14, prior to reimmunization, and day 21, one week after reimmunization, to measure ADAs. Measurement of serum ADAs showed a strong and significant reduction of anti-trastuzumab-deruxtecan IgG1 antibodies in anti-CD137-treated groups at both time-points (Fig. 7C), and a trend for a reduction in the IgG1 anti-trastuzumab levels (Fig. EV5B). The defect in ADA generation was accompanied by a decrease in long-lived bone marrow plasma cells (Fig. 7D).

Together, these findings demonstrate that agonistic anti-CD137 treatment can suppress B-cell responses not only in vaccination immune responses but also in pathological settings, such as tumor-associated TLSs and ADA formation during immunotherapies based on recombinant monoclonal antibodies.

## Discussion

We have mechanistically investigated the effects of exogenous CD137 agonism on B-cell responses elicited by vaccination and revealed the implications for tumor-associated tertiary lymphoid structures (TLSs) and for the generation of ADAs. We show that CD137 agonism disrupts germinal center (GC) responses regardless of the vaccination route and across secondary lymphoid organs and ectopic sites. In addition to GC abrogation, plasmablast (PB) formation is also reduced at later time-points, suggesting preferential impairment of GC-derived plasma cells rather than extrafollicular PBs, which is consistent with prior observations (Caloba et al, 2025). The GC defect coincides with diminished Tfh numbers, which are required for GC B-cell maturation. Whether impaired Tfh differentiation precedes or follows GC collapse induced by CD137 agonists warrants further studies. Importantly, those effects were observed using two different agonistic anti-CD137 antibodies binding to different epitopes.

We detected that CD137 is highly expressed on Tregs, Tfrs, and activated CD8^+^ T cells, and to a lesser extent on FDCs. Interestingly, we found that an anti-CD137 binds selectively to all these populations in vivo, including clear-cut binding to FDCs, even in spite of their lower CD137 levels of expression. This could be caused by the generation and binding of antibody-antigen immunocomplexes due to the presence of circulating soluble CD137 (Glez-Vaz et al, 2022). We actually observed that the FDC network expansion induced after immunization was curtailed by anti-CD137, albeit FDC organization at steady-state and function were largely preserved. FDCs expand from precursor proliferation in an LTα1β2- and TNFα-dependent manner (Le Hir et al, 1996; Pasparakis et al, 1996; Tumanov et al, 2004; Wang et al, 2001), with activated GC B cells being one of the major sources. It is therefore plausible that when GC B-cell activation is blunted, FDC remodeling does not further proceed. In line with this notion, FDC numbers in anti-CD137-treated, immunized mice resembled those in non-immunized controls. Importantly, their ability to capture and display antigens remained intact, arguing against FDC dysfunction as the primary mechanism of the GC B-cell defect induced by CD137 agonists.

We also excluded a role for CD8-dependent effector cytokines induced by CD137 agonism. IL-12 can favor extrafollicular over GC B-cell responses through multiple mechanisms, including suppression of Tfh differentiation, as reported in Toxoplasma and Salmonella models characterized by high IL-12 levels (Elsner and Shlomchik, 2019). In our case, despite observing reduced Tfh differentiation, IL-12 neutralization did not rescue GC formation.

Concomitant with GC disruption by CD137 agonists, we observed increased numbers of regulatory T cells (Tregs) and follicular regulatory T cells (Tfrs), likely driven by the enhanced proliferative capacity of both populations. This was accompanied by increased expression of the inhibitory receptor LAG-3. LAG-3 binds MHC class II molecules on target cells to dampen T-cell activation, and its ectopic expression has been shown to confer regulatory activity (Liang et al, 2008; Maruhashi et al, 2022). The increased immunosuppressive activity was also supported by the RNA-seq data, where we can see an increase in the expression of Granzyme-related genes and IL-1-regulation genes such as IL-1r2 and IL-1ra. Interestingly, the IL-1r2 gene is primarily expressed by Tfrs and has a decoy receptor function for IL-1, scavenging free IL-1 during inflammation. Mice deficient in IL-1r2 on Foxp3+ cells show an increased GC and TFH response (Pyrillou et al, 2024), indicating that the induction of this decoy receptor on Tfrs might be contributing to their immunosuppressive activity upon anti-CD137 stimulation. In addition, Granzyme B expression has been implicated in the suppression of T-helper cell responses during the GC reaction (Cao et al, 2007; Chen et al, 2025a), and TIGIT expression has been reported to upregulate Fgl2 on regulatory T cells (Joller et al, 2014), while its expression on Tfrs reportedly suppresses B-cell responses (Sungnak et al, 2020).

Although highly suppressive Tregs typically express elevated levels of CD25, Tfr cells residing within GCs generally display lower CD25 (IL2RA) expression, reflecting their activation and differentiation status (Jandl et al, 2017; Kumar et al, 2021; Wing et al, 2017). Consistent with this, our RNA-seq analysis revealed altered Il2ra expression in anti-CD137-treated mice, in agreement with the increased presence of GC-resident Tfr cells. This observation was further validated at the protein level by flow cytometry. Recent studies have shown that mature human Tfr cells express both ICOS and PD-1. However, we did not observe differences in the frequency of ICOS⁺PD-1⁺ Tfr cells following anti-CD137 treatment. In fact, PD-1+ expression on Tfrs has been reported to inhibit its suppression function (Sage et al, 2013). These findings may reflect species-specific differences between mice and humans or suggest that CD137 agonism primarily affects Tfr expansion and function rather than their maturation status. In contrast, we observed an increase in PD-1⁺CD25⁺ Treg populations, a phenotype frequently associated with activated and highly suppressive regulatory T cells.

Although our RNA-seq analysis was performed on bulk Foxp3-sorted cells, and Tfr and Treg populations may differ in the expression and function of specific molecules, including CD25 and PD-1, the transcriptional profile strongly supports a functional impact of CD137 agonism on Foxp3⁺ regulatory cells. Nevertheless, the precise mechanisms by which Tfr cells regulate B-cell responses and GC reactions remain incompletely understood. Current evidence suggests that both direct and indirect mechanisms contribute to this regulation, likely acting in concert, and additional pathways may remain to be identified.

Moreover, in our B–T co-culture system, Tregs from anti-CD137-treated mice more potently suppressed T-cell proliferation and B- and T-cell differentiation toward GC and Tfh phenotypes, respectively. However, future co-culture systems incorporating isolated Tfh, Tfr, and GC B cells could provide further insight into the specific immunosuppressive contribution of Tfr cells. Importantly, among T-cell subsets, Tregs and Tfrs displayed the brightest levels of CD137 expression and thereby anti-CD137 antibody capture in vivo. We observed that while the GC defect was extrinsic to direct anti-CD137 engagement on B cells, the effects on Treg were intrinsic.

In response to agonistic anti-CD137 mAb treatment, we observed an expansion of both Tregs and Tfr cells, likely driven by their increased proliferative capacity. However, the differentiation of Tfr cells from Treg precursors did not appear to be altered. Tfrs retain Foxp3 while expressing Tfh-related genes (e.g., Bcl6) and CXCR5, enabling access to B-cell follicles (Chung et al, 2011; Linterman et al, 2011; Wollenberg et al, 2011). Increased Tfr frequency has been linked to dampened GC responses and disruption of ongoing reactions (Jacobsen et al, 2021; Linterman et al, 2011), implicating Tfrs in anti-CD137–mediated GC abrogation. Notably, broad depletion of Foxp3⁺ cells partially restored GC responses in anti-CD137–treated mice, indicating that effects on Foxp3⁺ cells are a key but not exclusive component of the mechanism. Because our depletion strategy affected both Tfrs and conventional Tregs, it would be interesting to evaluate their independent contributions and identify additional mechanisms suggested by the experiments in bone marrow chimeric mice.

A number of CD137 agonists are progressing in their clinical development as cancer immunotherapy agents (Etxeberria et al, 2020). In this regard, it is of considerable relevance to realize that these agents may impact TLSs in the patients. In our mouse studies, we demonstrate that agonistic CD137 stimulation also disrupts ectopic B-cell responses in tumor-associated TLSs using a murine model of non-small cell lung cancer known to generate TLSs. Indeed, a striking abrogation of lung B-cell responses after anti-CD137 treatment was observed in this tumoral setting. Such a disruption likely follows the same Treg/Tfr-dependent mechanism as in SLOs, but this point remains to be formally determined. Given the fact that TLSs in cancer are favorable for the overall prognosis of cancer patients and for their clinical response to checkpoint inhibitors (Bouloudani et al, 2025), these findings hold most relevant therapeutic implications for combination immunotherapy strategies in oncology (Perez-Gracia et al, 2014; Sanmamed et al, 2022). It must be taken into account that in patients, TLSs are already present (or missing) at the time of using CD137 agonists against established. Hence, it remains to be seen what would be the effect of CD137 agonists on well pre-formed TLSs as opposed to disrupting their formation. Since our experiments could only be postponed for two weeks following intravenous tumor-cell inoculations, our results may fail to reflect what would happen in patients in whom tumors have evolved over long periods of time, forming TLSs for months or years.

Overall, it remains to be seen if the CD137 agonist effects are only pharmacological or whether they also take place as a result of the normal physiology of the CD137-CD137L axis. The effect of massively bioavailable CD137 agonists obviously differs from the function of a costimulatory pathway that operates on a cell-to-cell contact basis. In this line, although there are no reports on alterations of B-cell responses in CD137−/− or CD137L−/− mice, transgenic mice for 4-1BBL under the control of a MHC-II promoter experience a profound depletion and impairment of B-cell responses (Zhu et al, 2001). Moreover, CD137L−/− mice reportedly show a propensity to develop B-cell lymphomas (Ding et al, 2022; Middendorp et al, 2009). Therefore, the actual relevance of CD137/CD137L co-stimulation on the physiological functions of Tfr cells in health and disease is a subject that deserves future research.

ADAs, unfortunately, blight the efficacy of a number of immunoglobulin-based agents used in oncology (Paul et al, 2024), and even drastic means of pre-B-cell depletion have been tried (Peters et al, 2024). In a model of induced anti-drug antibodies (ADAs) against trastuzumab and trastuzumab-deruxtecan, anti-CD137 mAb reduced ADA levels and bone marrow plasma cells, supporting the rationale for making use of this anti-CD137 effect to mitigate ADA formation during specific antitumor combined regimens involving antibodies (Borregon et al, 2022). In this sense, constructs encompassing CD137 agonists may avoid generating antibodies directed to themselves.

All considered, we provide an in-depth characterization of how anti-CD137 agonists modulate humoral immunity in the context of GC reactions and offer mechanistic insight into how Tregs mediate such phenomena. These data are directly relevant to the design of rational combinations in which CD137 agonism might be paired with targeted therapies depleting or deactivating Tregs, if preservation of cancer-associated TLSs and humoral responses becomes our goal.

## Methods

**Table:** Reagents and tools table

Reagent/resource	Reference or source	Identifier or catalog number
**Experimental models**		
C57BL/6J	Inotiv	
CD137−/− (B6.Cg-Tnfrsf9tm1Byk)	Laboratory	B6.Cg-Tnfrsf9tm1Byk
CD45.1 (B6.SJL-Ptprca Pepcb/BoyJ)	Laboratory	B6.SJL-Ptprca Pepcb/BoyJ
Foxp3-DTR (B6.129(Cg)-Foxp3^tm3(HBEGF/GFP)Ayr^/J)	Laboratory	B6.129(Cg)-Foxp3^tm3(HBEGF/GFP)Ayr^/J
OT-II (B6.Cg-Tg(TcraTcrb)425Cbn/J)	Laboratory	B6.Cg-Tg(TcraTcrb)425Cbn/J
Foxp3-GFP hCD2-RFP	Laboratory (Veiga-Fernandes et al, 2007)	
**Antibodies**		
Purified anti-CD137	BioXCell	Clone 3H3
Purified anti-CD137	Bristol Myers (kind gift)	Clone 1D8
anti-IL12p40	BioXCell	Clone C17.8
anti-IFNγ	BioXCell	Clone XMG1.2
anti-CD8β	BioXCell	Clone Lyt3.2
InVivoMab polyclonal rat IgG Polyclonal	BioXCell	BE0094-25MG
CD45.2	BioLegend	Clone 104
Rabbit anti-PE	Rockland	cat. 100-4199
CD21/35	BioLegend	Clone 7E9
PDPN	BioLegend	Clone 8.1.1
MadCAM1	BD Biosciences	Clone MECA-367
CD31	BioLegend	Clone 390
CD95	BD Pharmigen	Clone Jo2
IgD	BioLegend	Clone 11-23c
GL7	BD Biosciences	GL7
CD45.1	BioLegend	Clone A20
CD138	BioLegend	Clone 281-2
CD38	BioLegend	Clone 90
Bcl6	BD Pharmigen	Clone K112-91
Foxp3	eBioscience	Clone FJK-16s
CD4	BioLegend	Clone GK1.5
CD25	BioLegend	Clone PC61
B220	BioLegend	Clone RA3-6B2
CD19	BioLegend	Clone 6D5
PD-1	BioLegend	Clone 29 F.1A12
CXCR5	BioLegend	Clone L138D7
ICOS	BD	Clone 7E.17G9
CD44	BD	Clone IM7
CD20	Abcam	Clone SP32
CD3	Abcam	Clone SP7
LAG-3	Biolegend	Clone C9B7W
CD8a	Biolegend	Clone 53-6.7
**Oligonucleotides and other sequence-based reagents**		
**Chemicals, enzymes and other reagents**		
SBA Clonotyping System HRP kit	Southern Biotech	5300-05
Alexa Fluor® 647 Protein Labeling Kit	Thermo	A20173
Ovalbumin	Invivogen	vac-pova
Trastuzumab (Herceptin)	Roche	45-2317
Trastuzumab-deruxtecan (Enhertu)	AstraZeneca	
Alhydrogel adjuvant 2%	Invivogen	vac-alu-50
R-Phycoerythrin	Thermo Fisher	P801
Baytril (Enrofloxacin)	Bayer	
Collagenase P	Roche	11213857001
DNase I	Sigma	11284932001
LIVE/DEAD™ Fixable Near IR (876) Viability Kit	Invitrogen	L34981
Click-iT EdU	Thermo Fisher	A10044
RNeasy Mini Kit	Qiagen	74136
CD4 Isolation Kit	Miltenyi Biotec	130-095-248
CD43 Microbeads	Miltenyi Biotec	130-049-801
RapiClear 1.52	Sunjilab	RC152001:10 ml
**Software**		
FlowJo 10	FlowJo	
GraphPad	Prism	V11
Qupath v0.4.4	https://qupath.github.io/	v0.4.4
Image J Fiji	https://imagej.net/ij/	
FlowJo	BD	v10
R / RStudio	R Foundation	
edgeR	Bioconductor (Chen, et al, 2025b)	
clusterProfiler	Bioconductor (Xu et al, 2024)	
msigdbr	Bioconductor (Liberzon et al, 2015)	
nf-core/rnaseq	nf-core	v3.21.0
**Other**		
CytoFLEX LX	Beckman Coulter	
LSM800 Confocal Microscope	Zeiss	
Bond RX Autostainer	Leica Biosystems	
PhenoImager HT	Akoya Biosciences	

### Mice

C57BL/6, CD137^−/−^ (B6.Cg-Tnfrsf9^tm1Byk^), CD45.1 (B6.SJL-Ptprca Pepcb/BoyJ), Foxp3-DTR (B6.129(Cg)-Foxp3^tm3(HBEGF/GFP)Ayr^/J), OT-II B6.Cg-Tg(TcraTcrb)425Cbn/J), and Foxp3-GFP hCD2-RFP (Veiga-Fernandes et al, 2007) mice were used. C57BL/6 mice were purchased from Inotiv. For all experiments, age- (7–12 weeks) and sex-matched animals were used. Mice were maintained under specific-pathogen-free conditions in the animal facility of the Cima Universidad de Navarra. All experimental procedures were approved by the Ethics Committee of the University of Navarra (CEEA 046-23 and CEEA 113-22) in accordance with European Council Guidelines.

For bone marrow chimeras, recipient mice were lethally irradiated with 9 Gy and reconstituted with 5×10⁶ donor bone marrow cells via intravenous injection the day after. Reconstituted mice received 0.2 mg/mL Baytril (Enrofloxacin) in drinking water for 2 weeks post-reconstitution.

For adoptive transfer experiments, splenocytes from WT or CD137⁻/⁻ mice were isolated, and 15 × 10⁶ cells were injected intravenously into CD45.1 recipient mice, which were immunized and treated the following day.

### Immunization

Mice were immunized with 10 μg of the antigen of interest (Ovalbumin, Trastuzumab, or Trastuzumab-Deruxtecan) in 100 μL mixed 1:1 with Alhydrogel adjuvant 2% (Invivogen) in the upper and lower flanks to target the brachial, axillary, and inguinal draining LNs. For intraperitoneal immunizations, 100 μg of the protein was used. Mice injected with Trastuzumab or Trastuzumab-Deruxtecan were reimmunized 14 days later with 10 μg/site.

For analyzing FDCs functionality, mice were immunized intraperitoneally with 2 mg anti-antigen antibody in 200 μL of PBS (Rabbit anti-B-Phycoerythrin, cat. 100-4199, Rockland). Eighteen hours later, mice were injected subcutaneously with 10 μg of fluorescent antigen (R-Phycoerythrin cat. P801, Thermo Fisher) in 100 μL mixed 1:1 with Alhydrogel adjuvant 2% (Invivogen).

For antibody treatments, a single dose of 100 μg of anti-CD137 (3H3 or 1D8 clone (Shuford et al, 1997)) was given intraperitoneally at day 0 or 4. AF-647-labeled anti-CD137 was generated using Alexa Fluor® 647 Protein Labeling Kit (A20173) according to the manufacturer’s instructions. Excess dye was removed using Amicon columns (Sigma; 10 K MWCO).

For depleting or neutralizing antibody treatments, intraperitoneal anti-IL12p40 (clone C17.8; 500 μg), anti-IFNg (clone XMG1.2; 200 μg), anti-CD8b (clone Lyt3.2; 100 μg), or their isotype rat IgG from BioXcell were given every 2/3 days.

For depletion of Foxp3-expressing cells, Foxp3-DTR mice were treated intraperitoneally with either 660 ng or 66 ng of diphtheria toxin (Sigma; D0564) every 2–3 days, starting 1 day before immunization. Draining lymph nodes were analyzed 7 days after immunization.

For testing the neutralizing ability of anti-IL12p40 (C17.8) and anti-IFNγ (XMG1.2), mice were injected intraperitoneally with 10 μg of recombinant IL-12 and treated with the neutralizing antibodies at days −1 and 1. Spleens and LNs were analyzed 24 h after IL-12 injection by flow cytometry.

Investigators were not blinded during animal treatment and sample collection.

### Isolation of cell subsets

For B-cell analysis, lymph nodes and spleens were harvested, homogenized through 40-μm strainers, and washed in PBS containing 2% FBS. Spleen cells were treated with ACK buffer (0.15 M NH₄Cl, 10 mM KHCO₃, 0.1 mM EDTA, pH 7.2–7.4) for 3 min to lyse erythrocytes and then washed in PBS + 2% FBS.

For FDCs preparation and lung tissue analysis, draining lymph nodes or lungs were disaggregated into small pieces with 25 G needles and collected in RPMI-1640 medium containing 2% FBS, 20 mM HEPES pH 7.2, 0.1 mg/ml collagenase P (Roche), and 25 μg/ml DNase I (Sigma). Dissociated tissue was incubated at 37 °C for 60 min, recollecting supernatant every 15 min. After enzymatic digestion, cell suspensions were filtered using a 100-μm strainer and washed with PBS containing 0.5% FBS and 10 mM EDTA. Cell suspensions were used directly for staining with antibodies.

For B-cell isolation, spleens were disaggregated using a 40-μm strainer and treated with ACK buffer for 5 min. Single-cell suspension was subsequently incubated with CD43 microbeads (Miltenyi Biotech), following the manufacturer’s instructions. For CD4 T cell isolation, a CD4 T cell isolation kit was used (Miltenyi Biotech) following the manufacturer’s instructions. For T regulatory cells isolation from Foxp3-GFP mice, purified CD4 T cells were sorted based on the expression of GFP.

### Flow cytometry

For flow cytometry staining, single-cell suspensions were incubated with Fixable Viability Dye (Invitrogen) for 15 min in ice-cold PBS. Cells were subsequently incubated with anti-CD16/32 and the appropriate antibodies for 20 min at 4 degrees in PBS containing 0.5% FBS and 10 mM EDTA for FDC analysis or PBS containing 0.5% FBS and 2 mM EDTA for B-cell analysis. The following antibodies were used: CD45.2 (104) Biolegend, CD21/35 (7E9) Biolegend, PDPN (8.1.1) Biolegend, Madcam1 (MECA-367) BD Biosciences, CD31 (390) Biolegend, CD95 (Jo2) BD Pharmigen, IgD (11-23c) Biolegend, T- and B-Cell activation antigen (GL7) BD Biosciences, CD45.1 (A20) Biolegend, CD138 (281-2) Biolegend, CD38 (90) Biolegend, Bcl6 (K112-91) BD Pharmigen, Foxp3 (FJK-16s), CD4 (GK1.5), CD25 (PC61), B220 (RA3-6B2) CD19 (6D5), PD-1 (29 F.1A12), CXCR5 (L138D7) from Biolegend, ICOS (7E.17G9) from BD. Cells were acquired on a CytoFLEX LX and analyzed using the FlowJo 10.

Samples were excluded only in cases of technical failure, such as low cell viability or insufficient event acquisition due to flow cytometer blockage. All remaining samples were included in the final analysis.

### RNA sequencing

Foxp3-GFP hCD2-RFP mice were injected intraperitoneally with 100 μg of anti-CD137 or its isotype control. Four days later, CD4 T cells from spleens and LNs were purified using the CD4 purification kit (130-095-248 Milteny Biotech), following the manufacturer’s instructions. Afterward, cells were labeled with Fixable Viability Dye (Invitrogen) for 15 min in PBS and sorted based on GFP+ and RFP+ expression. RNA from the sorted cells was obtained using the RNAesy Mini Kit (Qiagen) following the manufacturer’s instructions. RNA was sequenced by Dreamgenics (low-input library; 100 M reads).

Fastq files generated were preprocessed according to the nf-core/rnaseq pipeline v3.21.0. Pseudo-alignment and quantification were performed with Salmon, and the resulting raw counts matrix was analyzed with R on the RStudio software. Data were subjected to differential gene expression analysis through edgeR (Chen et al, 2025b), gene set enrichment analysis (GSEA) through clusterProfiler (Xu et al, 2024), and finally, an immunosuppressive score was generated based on the following genes: Tgfb1, Tgfb2, Tgfb3, Il10, Nt5e, Lag3, Pdcd1, Gzma, Gzmb, Gzmk, Ctla4, Prf1, Ebi3, Il12a, Nrn1, Il2ra, Entpd1, Lgals9, Tigit, Il1r2 and Il1rn. For GSEA, Hallmark and Gene Ontology pathways were used from the MSigDB (Molecular Signature DB) (Liberzon et al, 2015; Subramanian et al, 2005) obtained through the msigdbr package from R. All packages were obtained from the Bioconductor environment (v3.21).

### ADAs detection

Sera was collected from mice that were immunized s.c with Trastuzumab or Trastuzumab-Deruxtecan and treated with anti-CD137 or its isotype control at day 0 (before immunization, day 7 and 14. Mice were reimmunized and treated again with anti-CD137. Blood was also collected at day 21 (7 days post-reimmunization). Plate-bound Trastuzumab or Trastuzumab-Deruxtecan (20 μg ml^–1^) was used to measure antigen-specific antibodies. Class-switched serum immunoglobulin levels were detected using the SBA Clonotyping System HRP kit (Southern Biotech). Antibody levels were plotted over the background on day 0.

### Microscopy

For microscopy staining, LNs were incubated for 4 h with Antigenfix solution (Diapath) and washed and permeabilized in PBS containing 1% BSA, 1% normal mouse sera and 2% Triton X-100 for 24 h. LNs were incubated with the antibody mix in the permeabilization buffer for 3 days at 22 °C while shaking. Organs were subsequently washed in permeabilization buffer for 24 h and incubated with RapiClear solution (1.52 RIN) for 24 h at RT. Clarified organs were imaged and mounted in RapiClear solution using an LSM800 confocal microscope. Images were obtained using. Analysis was performed using FIJI.

Immunofluorescence staining and analysis was performed as previously described on a Bond RX autostainer (Abengozar-Muela et al, 2020; Lopez-Janeiro et al, 2022). Four-micron-thick FFPE tissue sections were deparaffinized (Bond DeWax, Leica Biosystems) and rehydrated per standard protocols. Antigen retrieval was performed with Bond Epitope Retrieval Solution 2 (ER2, product number AR9640 Leica Biosystems), followed by two sequential cycles of staining, with each cycle including a 30-minute combined block and primary antibody incubation (Akoya antibody diluent/block), followed by a secondary HRP-conjugated polymer. Signal amplification was achieved with TSA-Opal fluorophores. The primary antibodies and corresponding fluorophores are anti-CD3 (rabbit polyclonal, clone: SP7, dilution: 1:500, product number: ab16669; Abcam, RRID:AB_443425) in Opal 520; anti-CD20 (rabbit monoclonal, clone SP32, dilution: 1:1000, product number: ab64088; Abcam, RRID:AB_1139386) in Opal 690. Nuclei were counterstained with Spectral DAPI (Akoya Biosciences, FP1490) and the stained tissues were mounted with ProLong Diamond Antifade mounting medium (Thermo Fisher Scientific). Stained slides were scanned using the PhenoImager™ HT Automated Quantitative Pathology Imaging System (Akoya Biosciences). After image acquisition, unmixing of the spectral libraries was performed with inForm software (Akoya Biosciences). Unmixed images were then imported into the open-source digital pathology software QuPath version 0.4.4 for TLS quantification. TLSs were identified and counted if possessing organized CD3 (T cell) and CD20 (B-cell) regions.

### KPAR experiments

The KPAR cell line was generously provided by Julian Downward laboratory (Boumelha et al, 2022). Cells were cultured in DMEM with 10% FBS as previously described. Cells were tested negatively for mycoplasma. A total of 3 × 10⁵ cells were injected intravenously through the tail vein. Fourteen days post-injection, mice were treated with anti-CD137 (clone 3H3) or isotype control. Two weeks later, lungs were analyzed by flow cytometry and microscopy.

### Ovalbumin-specific antibody measurement

Sera was collected from mice that were immunized s.c with ovalbumin and treated with anti-CD137 or its isotype control at day 0. Blood was also collected at day 14. Plate-bound Ovalbumin (20 μg ml^–1^) was used to measure antigen-specific antibodies. Class-switched serum immunoglobulin levels were detected using the SBA Clonotyping System HRP kit (Southern Biotech). Antibody titers are given as log_2_(dilution) × 40. Positive values were defined as those 3 s.d. above the mean values of the negative controls. Antibody levels were also shown as serial serum dilutions.

### Edu in vivo proliferation assessment

Mice were immunized s.c. with ovalbumin and treated with anti-CD137 or its isotype control on day 0. On days 2, 4, and 7 post-immunization, mice were injected i.p. with 2 mg EdU (5-ethynyl-2′-deoxyuridine, A10044; Thermo) and culled 5 h later. Draining LNs were analyzed by flow cytometry, and the Click-iT EdU reaction was performed following the manufacturer’s instructions.

## Supplementary information

Peer Review File

Source data Fig. 1

Source data Fig. 2

Source data Fig. 3

Source data Fig. 4

Source data Fig. 5

Source data Fig. 6

Source data Fig. 7

Expanded View Figures

## Data Availability

Data generated from bulk RNA-seq were deposited on the Gene Expression Omnibus (GEO) database and can be obtained from the following accession code: GSE334650. The source data of this paper are collected in the following database record: biostudies:S-SCDT-10_1038-S44318-026-00900-2.
