# Supplementary material for: CD137 agonists in mice suppress germinal center responses by increasing the immunosuppressive activity of follicular regulatory T-cells
Source: EMBO J. 2026 Aug 25;45(20):7041–69. doi: 10.1038/s44318-026-00900-2 (PMC13672347; doi:10.1038/s44318-026-00900-2)
Supplement: Supplementary file 1 [file 44318_2026_900_MOESM1_ESM.pdf]

THE EMBO JOURNAL

CD137 agonists in mice suppress germinal center responses by increasing the immunosuppressive activity of follicular regulatory T-cells

Ana Martínez-Riaño, Raquel Cuesta, Dayanna Salinas, Nekane Soria, Raluca Alexandru, Paula Molero-Glez, David Ruiz-Guillamon, Arantza Azpilikueta, Carlos Luri-Rey, Álvaro Lopez-Janeiro, Carlos E de Andrea, María S. Aymerich, Pedro Berraondo, Alvaro Teijeira, and Ignacio Melero

Corresponding author(s): Ignacio Melero (imelero@unav.es), Ana Martínez-Riañom(ana.martinezriano@bio-gipuzkoa.eus)

Review Timeline:

Submission Date:	4th Jan 26
Editorial Decision:	13th Feb 26
Revision Received:	22nd Jun 26
Editorial Decision:	14th Jul 26
Revision Received:	27th Jul 26
Accepted:	29th Jul 26

Editor: Ioannis Papaioannou

Transaction Report:

(Note: With the exception of the correction of typographical or spelling errors that could be a source of ambiguity, letters and reports are not edited. Depending on transfer agreements, referee reports obtained elsewhere may or may not be included in this compilation. Referee reports are anonymous unless the Referee chooses to sign their reports.)

1st Editorial Decision **13th Feb 2026**

Dear Dr. Melero,

Thank you again for submitting your manuscript EMBOJ-2026-123462 for consideration by The EMBO Journal, and for your patience during peer review. Your manuscript has now been seen by three experts in the field, and we have received the complete set of their comments, which you can find appended below.

As you will see, the referees recognize that this is a compelling, novel, and interesting study addressing a relevant topic in immunology. While they are all rather supportive, they also identify several limitations and raise a number of concerns, while providing detailed and reasonable suggestions in their reports for strengthening the manuscript further and increasing its significance and impact on the field.

In light of this input, I would like to invite you to submit a thoroughly revised version of your manuscript taking the referees' suggestions on board, along with a detailed point-by-point response addressing all referees' comments. Please note that it is The EMBO Journal policy to allow only a single round of major revision, and acceptance of your manuscript will therefore depend on the completeness of your responses in this revised version. Given the nature of the raised concerns, I think that it would be useful to discuss your revision plan already during the initial phase of your revision. You are very welcome to share with me a draft point-by-point response/revision plan explaining if there are any points you do not agree with or cannot address. We could then arrange a video call to discuss the details further, if necessary.

We generally allow three months as standard revision time (12 May, 2026). As a matter of policy, competing manuscripts published during this period will not negatively impact our assessment of the conceptual advance presented by your study. However, we request that you contact us as soon as possible upon publication of any related work, to discuss how to proceed. Should you foresee a problem in meeting this three-month deadline, please let us know in advance and we will be able to grant an extension.

Please let me know if you have any questions or comments that you would like to discuss with me. Thank you for the opportunity to consider your work for publication in The EMBO Journal. I look forward to your revision.

Best regards,

Ioannis

Ioannis Papaioannou, PhD
Editor, The EMBO Journal
i.papaioannou@embojournal.org

*****
Instructions for preparing your revised manuscript
*****

1. When you are ready to submit the revision, please upload:

- A Word file of the manuscript text (including legends of main Figures, EV Figures and Tables). Please make sure that changes are highlighted (or "tracked") to be clearly visible.

- Individual production-quality figure files (one file per figure). When assembling your figures, please refer to our figure preparation guidelines in order to ensure proper formatting and readability in print as well as on screen:
https://bit.ly/EMBOPressFigurePreparationGuideline
If the data shown in a figure are obtained from n {less than or equal to} 2, please use scatter plots showing the individual data points.
Please also include scale bars in all microscopy images.
The following points must be specified in each figure legend:
i. the name of the statistical test used to generate error bars and P values
ii. the number (n) of independent experiments (please specify technical or biological replicates) underlying each data point (discussion of statistical methodology can be reported in the Materials and Methods section, but figure legends should contain a basic description of n, P, and the test applied)
iii. the nature of the bars and error bars (s.d., s.e.m.).
See also guidelines for figure legends: https://www.embopress.org/page/journal/14602075/authorguide#figureformat

- A point-by-point response to the referees' comments, with a detailed description of the changes made (as a word file). All referees' concerns must be fully addressed and their suggestions taken on board. When preparing your letter of response to the referees' comments, please bear in mind that this will form part of the Review Process File and will therefore be available online to the community. Please note that you have the possibility to opt out of the transparent process at any stage prior to publication by letting the editorial office know (contact@embojournal.org); if you do opt out, the Review Process File link will point to the following statement: "No Review Process File is available with this article, as the authors have chosen not to make the review process public in this case.". For more details on our Transparent Editorial Process, please visit our website: https://www.embopress.org/page/journal/14602075/authorguide#transparentprocess

- Expanded View (EV) files (replacing Supplementary Information) that are collapsible/expandable online. A maximum of 5 EV Figures can be typeset. EV Figures should be cited as "Figure EV1, Figure EV2" etc. in the text, and their respective legends should be included in the manuscript file after the legends of regular figures. See detailed instructions regarding Expanded View files here:
https://www.embopress.org/page/journal/14602075/authorguide#expandedview

- For the figures that you do NOT wish to display as Expanded View figures, they should be bundled together with their legends in a single PDF file called "Appendix", which should start with a short Table of Contents (including page numbers). Appendix figures should be referred to in the main text as: "Appendix Figure S1, Appendix Figure S2" etc. Please see detailed instructions here: https://www.embopress.org/page/journal/14602075/authorguide#expandedview

- A complete author checklist, which you can download from our author guidelines (https://www.embopress.org/page/journal/14602075/authorguide). Please note that the checklist will also be part of the Review Process File.

2. Please note that no statistics should be calculated and shown in Figures if n=2. Please also note that each p value should be reported as an exact value.

3. Before submitting your revision, primary datasets (and computer code, where appropriate) produced in this study need to be deposited in appropriate public databases (see https://www.embopress.org/page/journal/14602075/authorguide#dataavailability). In particular, all datasets such as DNA or RNA sequencing data, or mass spectrometry data generated in the study must be deposited in appropriate repositories. The accession numbers, database, and the specific URLs (links) should be listed in a formal "Data availability" section (placed after Methods), following the example below:

"The RNA-seq datasets produced in this study are available in the following database:
Gene Expression Omnibus GSE46843 (https://www.ncbi.nlm.nih.gov/geo/query/acc.cgi?acc=GSE46843)"

*** All links should resolve to a page where the data can be accessed. ***

*** Please remember to provide in the Data availability section of your revised manuscript reviewer passwords if the datasets are not yet public. ***

*** The Data Availability Section is restricted to new primary data that are part of this study. In case you have no data that require deposition in a public database, please state so instead of referring to the database: "Our study includes no data deposited in public repositories." under the heading "Data availability". ***

4. The materials and methods need to be described in the manuscript using our structured methods format, which is now required for all research articles. According to this format, the Methods section includes a single "Reagents and Tools Table" - listing key reagents, experimental models, software and relevant equipment including their sources and relevant identifiers- followed by a "Methods and Protocols" section describing the methods. Please download and fill our Reagents and Tools Table template (.docx), which you can find in our author guide: https://www.embopress.org/page/journal/14602075/authorguide#structuredmethods. When submitting your revised manuscript, please do not include the Reagents and Tools Table in the Methods section of the manuscript but instead upload it as a separate file choosing the file type "Reagent Table".

5. Please check that the title and the abstract of the manuscript are brief, yet explicit, even to non-specialists. The length of the title should not exceed 100 characters, and the abstract should be a single paragraph not exceeding 175 words.

6. Please also note our reference format: https://www.embopress.org/page/journal/14602075/authorguide#referencesformat.

7. At EMBO Press we ask authors to provide source data for the main manuscript figures. You will receive a separate email with instructions for providing source data with your revised manuscript, including how to upload and organize the files.

8. Please remember: digital image enhancement is acceptable practice, as long as it accurately represents the original data and conforms to community standards. If a figure has been subjected to significant electronic manipulation, this must be noted in the

figure legend or in the "Materials and Methods" section. The editors reserve the right to request original versions of figures and the original images that were used to assemble the figure.

9. Our journal encourages inclusion of data citations in the reference list to directly cite datasets that were obtained from public databases. Data citations in the article text are distinct from normal bibliographical citations and should directly link to the database records from which the data can be accessed. In the main text, data citations are formatted as follows: "Data ref: Smith et al, 2001" or "Data ref: NCBI Sequence Read Archive PRJNA342805, 2017". In the Reference list, data citations must be labeled with "[DATASET]". A data reference must provide the database name, accession number/identifiers, and a resolvable link to the landing page from which the data can be accessed at the end of the reference. Further instructions are available at: https://www.embopress.org/page/journal/14602075/authorguide#referencesformat.

10. We request authors to consider both actual and perceived competing interests. Please review our policy (https://www.embopress.org/page/journal/14602075/authorguide#conflictsofinterest) and update your competing interests statement if necessary. Please name this section 'Disclosure and competing interests statement' and place it after the Acknowledgements section.

11. Please note that all corresponding authors are required to provide an ORCID ID upon submission of a revised manuscript (https://orcid.org/). Please find instructions on how to link your ORCID ID to your account in our manuscript tracking system in our Author guidelines
(https://www.embopress.org/page/journal/14602075/authorguide#authorshipguidelines).

12. We use CRediT to specify the contributions of each author in the journal submission system. CRediT replaces the author contribution section, which should be removed from the manuscript. Please use the free text box to provide more detailed descriptions. See also guide to authors:
https://www.embopress.org/page/journal/14602075/authorguide#authorshipguidelines.

13. Further information is available in our Guide For Authors: https://www.embopress.org/page/journal/14602075/authorguide

14. We would also welcome the submission of cover suggestions or motifs to be used by our Graphics Illustrator in designing a cover.

Referee #1:

Review of EMBOJ-2026-123462, "Costimulation of follicular regulatory T cells with CD137 (4-1BB) agonists disrupts B-cell responses"

- general summary and opinion about the principal significance of the study, its questions and findings

In this manuscript the authors attempt to address the mechanistic basis for how anti-CD137 agonists can disrupt humoral immunity. To address this, they employ various models of immunisation (such as OVA) and study the impact of CD137 agonism on the multiple cell types potentially involved. They show that agonistic anti-CD137 antibodies target activated $CD8^+$ T cells, Tregs, follicular regulatory T cells (Tfrs), and FDCs and that CD137 agonism impairs GC reactions upon vaccination, impairing antibody production. They also show that anti-CD137 antibodies suppress ectopic B-cell responses in TLS of tumors. They go on to show that these effects are dependent upon $Foxp3^+$ cells, with anti-CD137 treatment increasing both the abundance and immunosuppressive activity of Tregs and Tfrs in their vaccination model.

The data are novel, interesting and address a longstanding immunological phenomenon. The results are in general clear, well described and presented with an easily to follow narrative.

My comments below i hope will serve to improve the paper

One potential limitation that all of the observed effects are demonstrated with a single antibody clone (3H3, a potent and known agonist). Therefore the data could potentially reflect an antibody clone-specific phenomenon. Did the authors evaluate other agonistic CD137 mAb?
Importantly the authors already discuss whether the observed effects relate to normal physiology or due to drug-mediated

pharmacology.

Similarly, previously the authors have elegantly shown that another CD137 antibody, 1D8 is to some extent toxic in mice, evoking hyper-inflammatory responses (especially liver inflammation) that are dose limited. To counter this the authors previously designed a masked/prodrug 1D8 molecule, leading to activation in only the right (in that case, tumor) location (Etxeberria et al pnas 2021). Did the authors attempt to exploit the same molecule in this study (in the TLS work). That would certainly increase the translational impact of the study - potentially providing a ready-made solution to any toxicity issues (which have so far limited CD137 agonists such as urelumab).

I could not readily discern from the figure legends how many times each experiment had been performed. In Figure 1, 4 data points presumably indicate 4 mice, but how many times was the experiment performed? Figure 2 shows 3 datapoints. Figure 4 by comparison shows >10 datapoints. Is this from >1 experiment? This (numbers of experiments as well as mice) should be detailed for all figures to evaluate reproducibility.

Of direct relevance, do the authors expect that they saw only limited defective plasmablast generation/ differentiation in anti-CD137-treated mice due to the relatively short time-frame of the study? Or was it simply that they had insufficient numbers of animals to conclude this (n=4)? See comment above
Data in some other figures lack routine statistical significance at $p<0.05$ due to small numbers.

- specific major concerns essential to be addressed to support the conclusions

Do the authors expect that they saw only limited defective plasmablast generation/ differentiation in anti-CD137-treated mice due to the relatively short time-frame of the study? Or was it simply that they had insufficient numbers of animals to conclude this (n=4)? See comment above

Perhaps the plots have been mixed in Figure 4A - the text reads "Fourteen days post-immunization, anti-CD137-treated mice displayed Tfh frequencies comparable to those of non-immunized controls and approximately two-fold decrease as compared with isotype-treated mice (Fig. 4A, Fig. EV3B)" and yet the %'s indicated on the representative plots show that the TFH increase with anti-CD137.

Did the authors evaluate the efficacy of the depleting/blocking antibodies used in Figure 5? i.e. assess controls as evidence that the CD8, IFNG and IL-12 mAbs had their desired effects? Particularly after immunisation this might be important.

- minor concerns that should be addressed
In Figure 2A was statistical analysis performed? This would complement the visual presentation

To appreciate better the data displayed in figure 2C, some raw flow cytometry plots comparing ex-vivo staining (day 0) versus day 1 might be helpful.

Some minor typos - e.g. line 402 "To explore further the possible effects on Tregs induced by the anti-CD137 agonism, Foxp3-GFP hCD2-RFP were" (missing the word mice or cells)

- any additional non-essential suggestions for improving the study (which will be at the author's/editor's discretion)

Possibly the white diagonal line for 3C is missing on the isotype magnification? Also the white box to show which area is being expanded

The IgD legend in figure 3C might be better placed to improve visualisation

Referee #2:

In this manuscript the authors assess how agonistic a4-1BB alters humoral immunity. The authors show that a4-1BB limits GC B cells, while simultaneously expanding Treg and Tfr cells, an effect that is not dependent on CD8s, IFNg or IL-12. Furthermore, RNAseq and functional experiments demonstrate that Treg from a4-1BB may be more activated with higher suppressive capacity. Lastly, the authors show that the decrease in B cell responses with a4-1BB can also occur in tumor derived TLS and antibody drug conjugates. Overall, this is a fairly compelling study on how a4-1BB controls humoral immunity. However, some claims are not substantiated with data presented and some data could be strengthened to enhance significance of the work.
Major points
1. This entire study was performed with the 3H3 a4-1BB clone. Key results (decrease in GC B, etc.) should be confirmed with a second a4-1BB agonistic antibody clone for confirmation.
2. A central claim (see title) is that a4-1BB affects Tfr cells both by expansion and suppressive function, but neither of these are

shown directly. Fig. 4c should show Tfr % of total Tregs, not of Tfh. This will help determine whether a4-1BB mostly affects Treg expansion and increased Tfr are due to increased precursors to Tfr, or whether Tfr differentiation is affected. Additional timepoints in Fig. 4 should also be included. Moreover, suppression assays in EV4e should be performed with sorted Tfr/Tfh/B in context of immunization to confirm whether Tfr cells have altered suppressive function.
3. Data in Fig. 6c-d indicate that a4-1bb still limits GC responses in the absence of Treg. There are no statistical increases between WT aCD137 and DTR aCD137, despite the authors claims. This should be strengthened.
4. OVA specific IgG should be assessed in serum in Figure 1 to substantiate the claim that a4-1BB enhances antigen specific responses.
5. FDC usually do not expand that much after immunization, so results in Fig. 3a are surprising. This is especially a concern since total FDC do not seem to be different by IF in Fig. 3c. Gating of FDC per condition should be shown for Fig. 3a.
6. The plots in Fig. 4a show increase in Tfh, but do not match up with the bar graphs.
7. Engraftment in Fig. 6a should be shown by flow cytometry. It is surprising that sufficient B and Treg can engraft in an intact host with total transfer.
8. Most Tfr references are over 15 years old, and newer references have further clarified Tfr differentiation and programming and should be cited (doi: 10.1038/s41590-025-02373-7).
Minor points
9. EV2c has multiple gates so it is unclear what is carried forward. Likewise, EV2d has CD4 on both axes.
10. Ref #40 shows that some Tfh can upregulate FoxP3 during GC contraction, however it is not clear if these are actually Tfr cells.
11. Line 337. It is suggested that Tfr suppress via PD-1, but PD-1 actually inhibits Tfr suppression (doi: 10.1038/ni.2496)

Referee #3:

The manuscript by Martinez-Riano and colleagues describes the impact of a CD137 (4-1BB) on the Tfh/Tfr cell balance, leading to disrupted humoral responses and GC/TLS formation. The phenotype that is observed with agonist CD137 antibodies is striking in its magnitude. Furthermore, the cellular circuits underlying the observed phenotype could be dissected using robust mouse models. Therefore, the conclusions are strongly supported by the experimental data, and the findings have clear clinical implications. There are some minor issues that could be addressed to improve the manuscript.

1. The magnitude of CD137 expression on Tfr and Treg cells represented in the histograms from Fig 2A is surprisingly low. It raises the question of whether the expression depends on an activation stage of these cells, which would suggest that the agonist antibody was acting primarily on Treg/Tfr cells engaged in the ongoing immune response rather than targeting all Treg/Tfr cells (with implications for biodistribution and off-target effects). The manuscript would benefit from information on the CD137 expression in Tfr/Treg at different stages of activation.

2. The immunosuppressive score, in Fig 6E and the companion text, may require further discussion. The expression of "characteristic" Treg-suppressive markers, most notably CD25 (IL2RA gene), changes significantly as Tfr cells mature (see Kumar Sci Immunol 2021). As such, it may constitute a better approach to treat Treg and Tfr "suppressive" signatures differently.

3. Related to the previous point, in addition to the numerical changes of Treg and Tfr cells, the authors could describe whether there are changes in the maturation of those cells, something that can be observed by flow cytometry (see, regarding Tfr maturation, Ribeiro Sci Adv 2025).

In conclusion, this manuscript provides compelling evidence for the impact of agonist anti-CD137 antibodies on humoral responses, dependent on Treg/Tfr cells. It is a major contribution to the research community with clear clinical implications.

Referee #1:

Review of EMBOJ-2026-123462, "Costimulation of follicular regulatory T cells with CD137 (4-1BB) agonists disrupts B-cell responses"

- general summary and opinion about the principal significance of the study, its questions and findings

In this manuscript the authors attempt to address the mechanistic basis for how anti-CD137 agonists can disrupt humoral immunity. To address this, they employ various models of immunisation (such as OVA) and study the impact of CD137 agonism on the multiple cell types potentially involved. They show that agonistic anti-CD137 antibodies target activated $CD8^+$ T cells, Tregs, follicular regulatory T cells (Tfrs), and FDCs and that CD137 agonism impairs GC reactions upon vaccination, impairing antibody production. They also show that anti-CD137 antibodies suppress ectopic B-cell responses in TLS of tumors. They go on to show that these effects are dependent upon $Foxp3^+$ cells, with anti-CD137 treatment increasing both the abundance and immunosuppressive activity of Tregs and Tfrs in their vaccination model.

The data are novel, interesting and address a longstanding immunological phenomenon. The results are in general clear, well described and presented with an easily to follow narrative.

Response 1:

Thank you for the overall positive appraisal of our manuscript and the detailed review.

My comments below i hope will serve to improve the paper

One potential limitation that all of the observed effects are demonstrated with a single antibody clone (3H3, a potent and known agonist). Therefore the data could potentially reflect an antibody clone-specific phenomenon. Did **the authors evaluate other agonistic CD137 mAb**? Importantly the authors already discuss whether the observed effects relate to normal physiology or due to drug-mediated pharmacology.

Response 2:

As suggested by the reviewer, we have evaluated the inhibition of GC and $IgG1^+$ class-switched responses using the agonistic anti-CD137 clone 1D8 (Fig. EV1E). In addition, we validated the observed reduction in Tfh differentiation and the increase in Treg and Tfr percentages using the 1D8 monoclonal antibody (Fig. EV3G). These results support and generalize the conclusion that the observed effects are associated with pharmacological CD137 activation rather than being specific to the 3H3 antibody.

Similarly, previously the authors have elegantly shown that another CD137 antibody, 1D8 is to some extent toxic in mice, evoking hyper-inflammatory responses (especially liver inflammation) that are dose limited. To counter this the authors previously designed a masked/prodrug 1D8 molecule, leading to activation in only the right (in that case, tumor) location (Etxeberria et al pnas 2021). **Did the authors attempt to exploit the same molecule in this study (in the TLS**

work). That would certainly increase the translational impact of the study - potentially providing a ready-made solution to any toxicity issues (which have so far limited CD137 agonists such as urelumab).

Response 3:

Unfortunately, we were unable to use the masked/prodrug version of the 1D8 molecule in the present study due to an expired contract with Cytomx and lack of interest from the new managers of the company. Nevertheless, we evaluated the impact of the unmodified 1D8 antibody on TLS development by treating KPAR-bearing mice with a single dose of 1D8 (100 µg) (Fig. EV5A). Under these conditions, we observed a reduction in intrapulmonary B cells and GC B cells, again supporting the notion that CD137 agonism impairs TLS-associated humoral responses with a different anti-CD137 antibody.

I could not readily discern from the **figure legends how many times each experiment had been performed**. In Figure 1, 4 data points presumably indicate 4 mice, but how many times was the experiment performed? Figure 2 shows 3 datapoints. Figure 4 by comparison shows >10 datapoints. Is this from >1 experiment? **This (numbers of experiments as well as mice) should be detailed for all figures to evaluate reproducibility**.

Response 4:

We thank the reviewer for this suggestion. We have now added detailed information regarding the number of independent experiments and biological replicates to all figure legends.

Each dot represents an independent mouse / biological sample.

Of direct relevance, do the authors expect that they saw only **limited defective plasmablast generation/ differentiation in anti-CD137-treated mice** due to the relatively short time-frame of the study? **Or was it simply that they had insufficient numbers of animals** to conclude this (n=4)? See comment above
Data in some other figures lack routine statistical significance at $p<0.05$ due to small numbers.

Response 5:

We analyzed plasmablast generation at later time points following immunization. While at day 10, we only observed a trend toward reduced plasmablast generation, by day 14 we detected a statistically significant decrease between groups (Fig. 1D, EV1 D). Furthermore, reduced plasma cells frequencies were also observed at day 21 in the bone marrow (Fig. 7D). We have now included the day 14 data in the revised manuscript to strengthen this conclusion with statistical significance.

- specific major concerns essential to be addressed to support the conclusions

Perhaps the **plots have been mixed in Figure 4A** - the text reads "Fourteen days post-immunization, anti-CD137-treated mice displayed Tfh frequencies comparable to those of non-immunized controls and approximately two-fold decrease as compared with isotype-treated mice (Fig. 4A, Fig. EV3B)" and yet the %'s indicated on the representative plots show that the TFH increase with anti-CD137.

Response 6:

We apologize for this error and we are immensely grateful for you having spotted it. The representative plots have now been changed accordingly.

Did the authors **<u>evaluate the efficacy of the depleting/blocking antibodies</u>** used in Figure 5? i.e. assess controls as evidence that the <u>CD8, IFNG and IL-12 mAbs had their desired effects</u>? Particularly after immunisation this might be important.

Response 7:

Although all antibodies used had been commercially purchased, we performed additional in vivo validation experiments as suggested by the reviewer (Fig. EV3J, K). To confirm CD8 depletion efficiency, lymph nodes were analyzed 24 h after treatment (Fig. EV3J). To validate IFNγ and IL-12 neutralization, mice were injected intraperitoneally with recombinant p70 IL-12 (10 µg), which increase MHC-I expression on dendritic cells in a manner at least partially dependent on IFNg. MHC-I expression levels on CD11c+ dendritic cells were then used as a functional readout for both stimulation and neutralization efficiency (Fig. EV3K).

- minor concerns that should be addressed

In <u>Figure 2A was statistical analysis performed</u>? This would complement the visual presentation

Response 8:

We have performed a one-way ANOVA test simply to state that there is variability in the level of CD137 expression on the indicated cell types.

To appreciate better the data displayed in figure 2C, some raw flow cytometry plots comparing ex-vivo staining (day 0) versus day 1 might be helpful.

Response 9:

The raw flow cytometry plots are presented in the figure EV2 F comparing the isotype negative control (grey) and the a-CD137 antibody (blue) at 24h.

Some **minor typos** - e.g. line 402 "To explore further the possible effects on Tregs induced by the anti-CD137 agonism, Foxp3-GFP hCD2-RFP were" (missing the word mice or cells)

Response 10:

We have already corrected it.

- any additional non-essential suggestions for improving the study (which will be at the author's/editor's discretion)

Possibly the white diagonal line for 3C is missing on the isotype magnification? Also the white box to show which area is being expanded

Response 11:

We have corrected it

The IgD legend in figure 3C might be better placed to improve visualisation

Response 12:

We have corrected it

Referee #2:

In this manuscript the authors assess how agonistic a4-1BB alters humoral immunity. The authors show that a4-1BB limits GC B cells, while simultaneously expanding Treg and Tfr cells, an effect that is not dependent on CD8s, IFNg or IL-12. Furthermore, RNAseq and functional experiments demonstrate that Treg from a4-1BB may be more activated with higher suppressive capacity. Lastly, the authors show that the decrease in B cell responses with a4-1BB can also occur in tumor derived TLS and antibody drug conjugates. Overall, this is a fairly compelling study on how a4-1BB controls humoral immunity. However, some claims are not substantiated with data presented and some data could be strengthened to enhance significance of the work.

Response 13:

We are very grateful for the encouraging comments and the detailed review of our work.

Major points

1. This entire study was performed with the 3H3 a4-1BB clone. Key results (decrease in GC B, etc.) should be **confirmed with a second a4-1BB agonistic antibody** clone for confirmation.

Response 14:

As suggested, we repeated key experiments using the agonistic anti-CD137 clone 1D8 (see response to reviewer 1). Specifically, we analyzed GC and IgG1^{+} class-switched responses (Fig. EV1E), as well as Tfh, Treg, and Tfr differentiation (Fig. EV3G). In addition, we confirmed the effects of CD137 agonism on TLS biology using the 1D8 clone (Fig. EV5A). Together, these data demonstrate that the observed effects are not restricted to the 3H3 clone.

2. A central claim (see title) is that **a4-1BB affects Tfr cells both by expansion and suppressive function, but neither of these are shown directly**. Fig. 4c should show Tfr % of total Tregs, not of Tfh. This will help determine whether a4-1BB mostly affects Treg expansion and increased Tfr are due to increased precursors to Tfr or whether Tfr differentiation is affected.

Additional timepoints in Fig. 4 should also be included

Moreover, **suppression assays in EV4e should be performed with sorted Tfr/Tfh/B in context of immunization** to confirm whether Tfr cells have altered suppressive function.

Response 15:

We have now reanalyzed the data as suggested and included Tfr frequencies within the total Treg compartment (Fig. 4E). These analyses indicate that the increase in Tfr cells is primarily driven by expansion of the Treg precursor pool rather than preferential differentiation into Tfr cells. This is now presented in results and discussed in the discussion section. We are grateful to the reviewer for having realized this matter.

In addition, we evaluated in vivo proliferation using EdU incorporation and found that anti-CD137 treatment increased proliferation of both Tregs and Tfrs (Fig. 6E), with a comparable ~3-fold increase in both populations.

We also included additional time points (days 4 and 7 post-immunization) analyzing Tfh, Tfr, and Treg frequencies, as well as Tfh/Tfr ratios (Fig. EV3E-F) in addition to day 14 on Figure 4.

Regarding functional suppression assays using sorted Tfr/Tfh/B-cell populations, we attempted multiple experimental attempts; however, the number of viable cells recovered after sorting was insufficient to establish reproducible in vitro assays. We have now acknowledged this technical limitation in the manuscript.

3. Data in Fig. 6c-d indicate that a4-1bb still limits GC responses in the absence of Treg. **There are no statistical increases between WT aCD137 and DTR aCD137**, despite the authors claims. This should be strengthened.

Response 16:

We appreciate this comment. As noted by the reviewer, the initial dataset lacked statistical significance due to variability among animals. We have now added an additional cohort of mice, increased the sample size and thereby reduced variability, which allowed us to reach statistical significance (Figure 6C).

4. OVA specific IgG should be assessed in serum in Figure 1 to substantiate the claim that a4-1BB enhances antigen specific responses.

Response 17:

We performed ELISA analyses as suggested by the reviewer (Fig. 1F). These experiments revealed a significant reduction in OVA-specific IgG1 titers 14 days after immunization in anti-CD137-treated mice, in agreement with reduced B-cell responses.

5. FDC usually do not expand that much after immunization, so results in Fig. 3a are surprising. This is especially a concern **since total FDC do not seem to be different by IF in Fig. 3c. Gating of FDC per condition should be shown for Fig. 3a.**

Response 18:

We have now included the gating strategies used for these analyses (Fig. EV3C). Importantly, previous studies have shown that immunization can induce FDC expansion at magnitudes comparable to those observed here (PMID: 37443283). In addition, we analyzed non-immunized mice treated with isotype or anti-CD137 agonistic antibody and observed no differences in FDC frequencies five days later (Figure 3A). These findings support the idea that the effects of anti-CD137 on FDCs are indirect and driven by the altered immune response rather than direct CD137 targeting of FDCs.

6. The **plots in Fig. 4a** show increase in Tfh, but do not match up with the bar graphs.

Response 19:

Thank you very much for the correction, we apologize for the copy-paste mistake.

7. **Engraftment in Fig. 6a should be shown by flow cytometry**. It is surprising that sufficient B and Treg can engraft in an intact host with total transfer.

Response 20:

We have now included flow cytometric analyses of donor-cell engraftment (CD45.2⁺ cells) in recipient mice (Fig. EV4B). As correctly indicated by the reviewer, engraftment levels were relatively low. Consequently, large numbers of events had to be acquired to obtain reproducible analyses of GC B cells and Treg populations. Unfortunately, Tfr cell numbers remained too low for reliable quantification and those data cannot be included.

8. **Most Tfr references are over 15 years old**, and newer references have further clarified Tfr differentiation and programming and should be cited (doi: 10.1038/s41590-025-02373-7).

Response 21:

We have updated the references as suggested by the reviewer.

Minor points

9. **EV2c has multiple gates so it is unclear what is carried forward**. Likewise, **EV2d has CD4 on both axes**.

Response 22:

Thank you very much for this point, we have clarified the explanations and corrected the gating strategy display.

10. Ref #40 shows that some Tfh can upregulate FoxP3 during GC contraction, however it is not clear if these are actually Tfr cells.

Response 23:

We agree with this limitation of the aluded reference since the immunosuppressive ability of those newly generated Foxp3+ Bcl6+ cells is not shown. However, their presence correlates with the GC shutdown period and such lymphocytes express the two master regulators that define TFRs. We briefly acknowledge such limitation in discussion.

11. Line 337. It is suggested that Tfr suppress via PD-1, but PD-1 actually inhibits Tfr suppression (doi: 10.1038/ni.2496)

Response 24:

We have modified the text according to the published data and provided the reference.

Referee #3:

The manuscript by Martinez-Riano and colleagues describes the impact of a CD137 (4-1BB) on the Tfh/Tfr cell balance, leading to disrupted humoral responses and GC/TLS formation. The phenotype that is observed with agonist CD137 antibodies is striking in its magnitude. Furthermore, the cellular circuits underlying the observed phenotype could be dissected using robust mouse models. Therefore, the conclusions are strongly supported by the experimental data, and the findings have clear clinical implications. There are some minor issues that could be addressed to improve the manuscript.

1. **The magnitude of CD137 expression on Tfr and Treg cells represented in the histograms from Fig 2A is surprisingly low**. It raises the question of whether the expression depends on an activation stage of these cells, which would suggest that the agonist antibody was acting primarily on Treg/Tfr cells engaged in the ongoing immune response rather than targeting all Treg/Tfr cells (with implications for biodistribution and off-target effects). **The manuscript would benefit from information on the CD137 expression in Tfr/Treg at different stages of activation.**

Response 25:

We appreciate this observation. We have now included an additional independent experiment in Figure 2A and expanded the analysis to days 4 and 7 post-immunization (Fig. EV2E). To facilitate comparisons across populations, CD137 expression is now represented as fold-change over FMO controls. What cannot be addressed is if the level of CD137 expression beyond a detectable threshold has differential functional effects. This is a topic that we are addressing with regard to CD137-based cancer immunotherapy and it is a complex issue.

2. The **immunosuppressive score, in Fig 6E** and the companion text, may **require further discussion**. The expression of "characteristic" Treg-suppressive markers, most notably CD25 (IL2RA gene), changes significantly as Tfr cells mature (see Kumar Sci Immunol 2021). As such, it may constitute a better approach to treat Treg and Tfr "suppressive" signatures differently.

Response 26:

We have expanded the discussion regarding the immunosuppressive score and added the references used to generate the gene signature. We agree with the reviewer that suppressive programs in Tregs and Tfrs may not fully overlap and therefore we now discuss this limitation more explicitly.

3. Related to the previous point, in addition to the numerical changes of Treg and Tfr cells, the **authors could describe whether there are changes in the maturation of those cells**, something that can be observed by flow cytometry (see, regarding Tfr maturation, Ribeiro Sci Adv 2025).

Response 27:

As suggested, we further characterized Treg and Tfr maturation markers (Fig. EV3H-I). We observed that Tfrs from anti-CD137-treated mice displayed lower CD25 surface expression, consistent with a GC-localized phenotype. However, we did not observe differences in double-positive ICOS+PD1+ cells. These discrepancies may reflect species-specific differences or indicate that CD137 agonism does not substantially alter Tfr maturation. In contrast, we observed an increase in highly suppressive PD-1^{+}CD25^{+} Treg populations.

In conclusion, this manuscript provides compelling evidence for the impact of agonist anti-CD137 antibodies on humoral responses, dependent on Treg/Tfr cells. It is a major contribution to the research community with clear clinical implications.

Response 28:

We are very grateful for the positive appraisal of our work.

1st Revision - Editorial Decision 14th Jul 2026

Dear Dr. Melero,

Thank you for the submission of your revised manuscript (EMBOJ-2026-123462R) to The EMBO Journal for our consideration, and for your patience during re-review. Your manuscript and your point-by-point responses to the previous comments of the referees have now been seen by them, and we have received their new reports, which you can find appended below.

I am pleased to say that, as you will see, the referees find the manuscript strengthened and substantially improved, and all previously raised concerns adequately addressed. They now all recommend publication of the manuscript in The EMBO Journal. In light of this expert input, I am pleased to let you know that your manuscript has been accepted in principle for publication in our journal.

Before we can move forward with the next steps towards publication of your article, there are a few changes and corrections from the editorial side we need you to make in a final version of the manuscript:

- Thank you for depositing your RNA-sequencing data to the Gene Expression Omnibus (GEO) database. Please make sure that these data will be publicly available at the time of publication. The reviewer token can now be removed from the Data Availability statement of the manuscript.

- Please also provide in the Data Availability statement an ID and URL to the Source Data deposited in BioStudies. If this is no longer relevant -as we note that you have already uploaded Source Data for your Figures to our online system- please remove the sentence referring to BioStudies from this section.

- Please note that the References format needs to be updated to the style used by The EMBO Journal. In particular, the list of references should be alphabetical (not numerical), and "et al." must be used after the names of the first 10 co-authors of each citation. Please check our author guidelines for more information on our format:
https://link.springer.com/journal/44318/submission-guidelines#cms-Reference-guidelines.

- All funding information provided in the Acknowledgements section of the manuscript must also be entered in our online system; currently, the following items are missing from the system:
"the Spanish Research Agency (MICIU/AEI) grant (ID: PID2024-156660OA-100) and the Ramón y Cajal grant (RYC2023-042880-I)".

- Please note that EMBO press papers are accompanied online by:
A) a short (2 sentences) summary of the findings and their significance,
B) 2-5 short bullet points highlighting the key results, and
C) a synopsis image in .jpg or .png format that is exactly 550 pixels wide and 300 pixels high. Please note that all text needs to be legible at the final size.
Please upload this information along with your revised manuscript (the text for A and B should be provided in a separate Word file).

- Thank you for uploading and correctly reorganizing the Source Data for your Figures. Please note that these must be accompanied by a completed Source Data checklist that our editorial staff has already requested. Please let our team know if you have any questions about this checklist.

- Please remove section "Summary" from the title page of the manuscript. This text could be used, after some textual revision, as the 2-sentence summary of the findings and their significance (please see above for more detailed instructions).

- Our data editors have checked your Figures and their legends, and raised the following queries. Please address their request below completely in your revised manuscript (all changes should be highlighted or "tracked"):
Please provide the exact p-values in the legends of Figures 1B, C; 2A, C; 4C, D; 5D, E; 6A, EV1 C, D; EV3 F, EV4 A, C, G.

- Manuscript sections need to be named and ordered as follows: Title page - Abstract - Introduction - Results - Discussion - Methods - Data Availability - Acknowledgements - Disclosure and Competing Interests Statement - References - Figure Legends - main Tables (if there are any) - Expanded View Figure Legends (if there are any).

- Please also note that as part of the EMBO Press transparent editorial process, The EMBO Journal publishes online a Peer Review File along with each accepted manuscript. This File will be published in conjunction with your paper and will include the referee reports, your point-by-point responses and all pertinent correspondence relating to the manuscript. Your Author's Checklist will also be published at the end of the Peer Review File. Please let us know in case you want to remove any data or figures from your point-by-point responses before they are published as part of the Peer Review File. Retaining unpublished data in the Peer Review File means that these count as published and that the Peer Review File would need to be referenced in future publications. Please let the editorial office know in case you want to remove any data from this file

(contact@embojournal.org).

We look forward to seeing a final version of your manuscript as soon as possible. Please let us know if you have any questions and use this link to submit your revision: xxxxx

Best regards,

Ioannis

Ioannis Papaioannou, PhD
Editor, The EMBO Journal
i.papaioannou@embojournal.org

Referee #1:

The authors have addressed my earlier concerns, strengthening the data and conclusions.

Referee #2:

The authors have addressed my concerns.

Referee #3:

The revised version of the manuscript addressed all the issues raised in the initial report. The manuscript is substantially improved and will be of great value to the community.

All minor editorial requests have been addressed by the authors.

In conclusion, this manuscript provides compelling evidence for the impact of agonist anti-CD137 antibodies on humoral responses, dependent on Treg/Tfr cells. It is a major contribution to the research community with clear clinical implications.

Response 28:

We are very grateful for the positive appraisal of our work.

2nd Revision - Editorial Decision 29th Jul 2026

Dear Ignacio,

Congratulations on an excellent manuscript! I am very pleased to inform you that it has been accepted for publication in The EMBO Journal. Thank you for comprehensively addressing the initially raised referees' concerns and our editorial requests for corrections and changes.

Your manuscript will be processed for publication by EMBO Press. It will be copy edited and you will receive page proofs prior to publication. Please note that you will be contacted by Springer Nature Author Services to complete licensing and payment information.

You may qualify for financial assistance for your publication charges - either via a Springer Nature fully open access agreement or an EMBO initiative. Check your eligibility: https://link.springer.com/journal/44318/how-to-publish-with-us

Should you be planning a Press Release on your article, please notify the production team via this form in order to coordinate publication and release dates: https://forms.cloud.microsoft/e/eH4A8JVP6g

If you have any questions, please do not hesitate to contact the Editorial Office. Thank you for your contribution to The EMBO Journal. Working with you has been a pleasure.

Best regards,

Ioannis

Ioannis Papaioannou, PhD
Senior Editor, The EMBO Journal - EMBO Reports - Molecular Systems Biology
i.papaioannou@embojournal.org

*****
Please note that it is The EMBO Journal policy for the transcript of the editorial process (containing referee reports and your response letters) to be published as an online supplement to each paper. If you should prefer removal of any referee-only figures included in the point-by-point response(s), e.g. because they may still be used for future publication or because they have been reproduced from published work by others, please do let us know immediately via response email.
More information is available here: https://link.springer.com/partners/embo-press/editorial-policies#Peer%20review

** Click here to be directed to your login page: https://emboj.msubmit.net

EMBO Press Author Checklist

Corresponding Author Name: Ana Martínez Riano & Ignacio Melero	
Journal Submitted to: EMBO Journal	
Manuscript Number: EMBOJ-2026-123462	

USEFUL LINKS FOR COMPLETING THIS FORM
The EMBO Journal - Author Guidelines
EMBO Reports - Author Guidelines
Molecular Systems Biology - Author Guidelines
EMBO Molecular Medicine - Author Guidelines

Reporting Checklist for Life Science Articles (updated January
This checklist is adapted from Materials Design Analysis Reporting (MDAR) Checklist for Authors. MDAR establishes a minimum set of requirements in transparent reporting in the life sciences (see Statement of Task: 10.31222/osf.io/9sm4x). Please follow the journal's guidelines in preparing your
Please note that a copy of this checklist will be published alongside your article.

Abridged guidelines for figures
1. Data
The data shown in figures should satisfy the following conditions:
- the data were obtained and processed according to the field's best practice and are presented to reflect the results of the experiments in an accurate and unbiased manner.
- ideally, figure panels should include only measurements that are directly comparable to each other and obtained with the same assay.
- plots include clearly labeled error bars for independent experiments and sample sizes. Unless justified, error bars should not be shown for technical
- if n<5, the individual data points from each experiment should be plotted. Any statistical test employed should be justified.
- Source Data should be included to report the data underlying figures according to the guidelines set out in the authorship guidelines on Data

2. Captions
Each figure caption should contain the following information, for each panel where they are relevant:
- a specification of the experimental system investigated (eg cell line, species name).
- the assay(s) and method(s) used to carry out the reported observations and measurements.
- an explicit mention of the biological and chemical entity(ies) that are being measured.
- an explicit mention of the biological and chemical entity(ies) that are altered/varied/perturbed in a controlled manner.
- the exact sample size (n) for each experimental group/condition, given as a number, not a range;
- a description of the sample collection allowing the reader to understand whether the samples represent technical or biological replicates (including how many animals, litters, cultures, etc.).
- a statement of how many times the experiment shown was independently replicated in the laboratory.
- definitions of statistical methods and measures:
  - common tests, such as t-test (please specify whether paired vs. unpaired), simple χ2 tests, Wilcoxon and Mann-Whitney tests, can be unambiguously identified by name only, but more complex techniques should be described in the methods section;
  - are tests one-sided or two-sided?
  - are there adjustments for multiple comparisons?
  - exact statistical test results, e.g., P values = x but not P values < x;
  - definition of 'center values' as median or average;
  - definition of error bars as s.d. or s.e.m.

Please complete ALL of the questions below.
Select "Not Applicable" only when the requested information is not relevant for your study.

Materials

Newly Created Materials	Information included in the manuscript?	In which section is the information available? (Reagents and Tools Table, Materials and Methods, Figures, Data Availability Section)
New materials and reagents need to be available; do any restrictions apply?	Yes	The informaion is included in the Data Availability section

Antibodies	Information included in the manuscript?	In which section is the information available? (Reagents and Tools Table, Materials and Methods, Figures, Data Availability Section)
For **antibodies** provide the following information:<br>- Commercial antibodies: RRID (if possible) or supplier name, catalogue number and or/clone number<br>- Non-commercial: RRID or citation	Yes	Information included in the Reagents and Tools table and in the Materials and Methods.

DNA and RNA sequences	Information included in the manuscript?	In which section is the information available? (Reagents and Tools Table, Materials and Methods, Figures, Data Availability Section)
**Short novel DNA or RNA including primers, probes**: provide the sequences.	Not Applicable	

Cell materials	Information included in the manuscript?	In which section is the information available? (Reagents and Tools Table, Materials and Methods, Figures, Data Availability Section)
**Cell lines:** Provide species information, strain. Provide accession number in repository **OR** supplier name, catalog number, clone number, and/**OR** RRID.	Yes	Information included in the Materials and Methods.
**Primary cultures:** Provide species, strain, sex of origin, genetic modification status.	Not Applicable	
Report if the cell lines were recently **authenticated** (e.g., by STR profiling) and tested for mycoplasma contamination.	Yes	Information included in the Materials and Methods.

Experimental animals	Information included in the manuscript?	In which section is the information available? (Reagents and Tools Table, Materials and Methods, Figures, Data Availability Section)
**Laboratory animals or Model organisms:** Provide species, strain, sex, age, genetic modification status. Provide accession number in repository **OR** supplier name, catalog number, clone number, **OR** RRID.	Yes	Information included in the Reagents and Tools table and in the Materials and Methods.
**Animal observed in or captured from the field:** Provide species, sex, and age where possible.	Not Applicable	
Please detail **housing and husbandry conditions**.	Yes	Information included in the Materials and Methods.

Plants and microbes	Information included in the manuscript?	In which section is the information available? (Reagents and Tools Table, Materials and Methods, Figures, Data Availability Section)
**Plants:** provide species and strain, ecotype and cultivar where relevant, unique accession number if available, and source (including location for collected wild specimens).	Not Applicable	
**Microbes:** provide species and strain, unique accession number if available, and source.	Not Applicable	

Human research participants	Information included in the manuscript?	In which section is the information available? (Reagents and Tools Table, Materials and Methods, Figures, Data Availability Section)
If collected and within the bounds of privacy constraints report on age, sex and gender or ethnicity for all study participants.	Not Applicable	

Core facilities	Information included in the manuscript?	In which section is the information available? (Reagents and Tools Table, Materials and Methods, Figures, Data Availability Section)
If your work benefited from core facilities, was their service mentioned in the acknowledgments section?	Yes	Acknowledgement section

Design

Study protocol	Information included in the manuscript?	In which section is the information available? (Reagents and Tools Table, Materials and Methods, Figures, Data Availability Section)
If study protocol has been **pre-registered, provide DOI in the manuscript**. For clinical trials, provide the trial registration number **OR** cite DOI.	Not Applicable	
Report the **clinical trial registration number** (at ClinicalTrials.gov or equivalent), where applicable.	Not Applicable	

Laboratory protocol	Information included in the manuscript?	In which section is the information available? (Reagents and Tools Table, Materials and Methods, Figures, Data Availability Section)
Provide DOI OR other citation details if **external detailed step-by-step protocols** are available.	Not Applicable	

Experimental study design and statistics	Information included in the manuscript?	In which section is the information available? (Reagents and Tools Table, Materials and Methods, Figures, Data Availability Section)
Include a statement about **sample size** estimate even if no statistical methods were used.	Yes	Information included in the Figure legends.
Were any steps taken to minimize the effects of subjective bias when allocating animals/samples to treatment (e.g. **randomization procedure**)? If yes, have they been described?	Not Applicable	
Include a statement about **blinding** even if no blinding was done.	Yes	Information included in the Materials and Methods.
Describe **inclusion/exclusion criteria** if samples or animals were excluded from the analysis. Were the criteria pre-established?<br><br>If sample or data points were omitted from analysis, report if this was due to attrition or intentional exclusion and provide justification.	Yes	Information included in the Materials and Methods.
For every figure, are **statistical tests** justified as appropriate? Do the data meet the assumptions of the tests (e.g., normal distribution)? Describe any methods used to assess it. Is there an estimate of variation within each group of data? Is the variance similar between the groups that are being statistically compared?	Yes	Information included in the Figure legends.

Sample definition and in-laboratory replication	Information included in the manuscript?	In which section is the information available? (Reagents and Tools Table, Materials and Methods, Figures, Data Availability Section)
In the figure legends: state number of times the experiment was **replicated** in laboratory.	Yes	Information included in the Figure legends.
In the figure legends: define whether data describe **technical or biological replicates**.	Yes	Information included in the Figure legends.

Ethics

Ethics	Information included in the manuscript?	In which section is the information available? (Reagents and Tools Table, Materials and Methods, Figures, Data Availability Section)
Studies involving **human participants**: State details of **authority granting ethics approval** (IRB or equivalent committee(s), provide reference number for approval.	Not Applicable	
Studies involving **human participants**: Include a statement confirming that **informed consent** was obtained from all subjects and that the experiments conformed to the principles set out in the WMA Declaration of Helsinki and the Department of Health and Human Services Belmont Report.	Not Applicable	
Studies involving **human participants:** For publication of **patient photos**, include a statement confirming that consent to publish was obtained.	Not Applicable	
Studies involving experimental **animals**: State details of **authority granting ethics approval** (IRB or equivalent committee(s), provide reference number for approval. Include a statement of compliance with ethical regulations.	Yes	Information included in the Materials and Methods.
Studies involving **specimen and field samples:** State if relevant **permits** obtained, provide details of authority approving study; if none were required, explain why.	Not Applicable	

Dual Use Research of Concern (DURC)	Information included in the manuscript?	In which section is the information available? (Reagents and Tools Table, Materials and Methods, Figures, Data Availability Section)
Could your study fall under dual use research restrictions? Please check biosecurity documents and list of **select agents and toxins** (CDC): https://www.selectagents.gov/sat/list.htm	Not Applicable	
If you used a select agent, is the security level of the lab appropriate and reported in the manuscript?	Not Applicable	
If a study is subject to dual use research of concern regulations, is the name of the **authority granting approval and reference number** for the regulatory approval provided in the manuscript?	Not Applicable	

Reporting

The MDAR framework recommends adoption of discipline-specific guidelines, established and endorsed through community initiatives. Journals have their own policy about requiring specific guidelines and recommendations to complement MDAR.

Adherence to community standards	Information included in the manuscript?	In which section is the information available? (Reagents and Tools Table, Materials and Methods, Figures, Data Availability Section)
State if relevant guidelines or checklists (e.g., **ICMJE, MIBBI, ARRIVE, PRISMA**) have been followed or provided.	Not Applicable	
For **tumor marker prognostic studies,** we recommend that you follow the **REMARK** reporting guidelines (see link list at top right). See author guidelines, under 'Reporting Guidelines'. Please confirm you have followed these guidelines.	Not Applicable	
For **phase II and III randomized controlled trials**, please refer to the **CONSORT** flow diagram (see link list at top right) and submit the CONSORT checklist (see link list at top right) with your submission. See author guidelines, under 'Reporting Guidelines'. Please confirm you have submitted this list.	Not Applicable	

Data Availability

Data availability	Information included in the manuscript?	In which section is the information available? (Reagents and Tools Table, Materials and Methods, Figures, Data Availability Section)
Have **primary datasets** been deposited according to the journal's guidelines (see 'Data Deposition' section) and the respective accession numbers provided in the Data Availability Section?	Yes	Information included in the Data Availability Section.
Were **human clinical and genomic datasets** deposited in a public access-controlled repository in accordance to ethical obligations to the patients and to the applicable consent agreement?	Yes	Information included in the Data Availability Section.
Are **computational models** that are central and integral to a study available without restrictions in a machine-readable form? Were the relevant accession numbers or links provided?	Not Applicable	
If publicly available data were reused, provide the respective **data citations in the reference list**.	Not Applicable	
