# Supplementary material for: CD137 agonists in mice suppress germinal center responses by increasing the immunosuppressive activity of follicular regulatory T-cells
Source: EMBO J. 2026 Aug 25;45(20):7041–69. doi: 10.1038/s44318-026-00900-2 (PMC13672347; doi:10.1038/s44318-026-00900-2)
Supplement: Supplementary file 2 [file 44318_2026_900_MOESM9_ESM.pdf]

Expanded View Figures

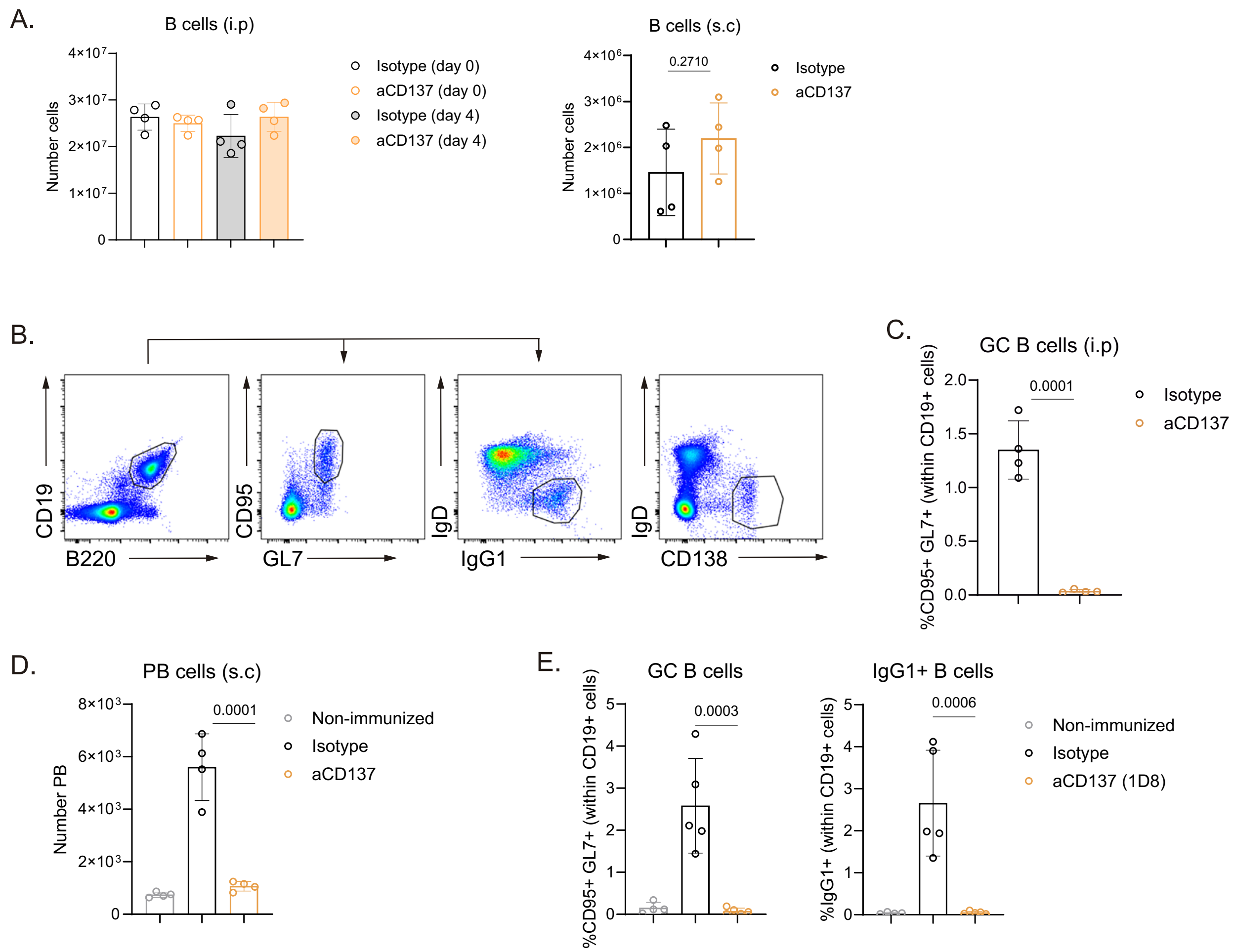

Figure EV1. Methodology and analysis of B-cell responses upon anti-CD137 treatment.

(**A**) Number of B cells in the spleen (left) or dLNs (right) of mice immunized with ovalbumin mixed with Alhydrogel intraperitoneally (100 µg) or subcutaneously (10 µg/site). Mice immunized intraperitoneally were treated at day 0 or 4 with anti-CD137 or its isotype control (100 µg i.p). Mice immunized subcutaneously were treated at day 0 with anti-CD137 or its isotype control (100 µg i.p) ($n = 4$ mice; representative of two independent experiments). (**B**) Flow cytometry gating strategy for B cells, GC B cells, IgG1 CS B cells and PB. (**C**) Percentage of GC B cells in the spleen of mice immunized i.p with ovalbumin mixed with Alhydrogel and treated at day 4 with anti-CD137 or its isotype control (100 µg i.p) ($n = 4$ mice; representative of two independent experiments). (**D**) Number of PB in the dLNs of non-immunized or immunized mice treated at day 0 with anti-CD137 or its isotype control (100 µg i.p) ($n = 4$ mice). (**E**) Percentage of GC B cells (left graph) and IgG1+ B cells (right graph) in non-immunized or immunized mice treated with isotype or anti-CD137 1D8 mAb ($n = 5$ mice; 1 independent experiment). All quantitative data were shown as means ± SD and analyzed using one-way ANOVA with multiple comparisons or *t*-tests.

 © The Author(s)

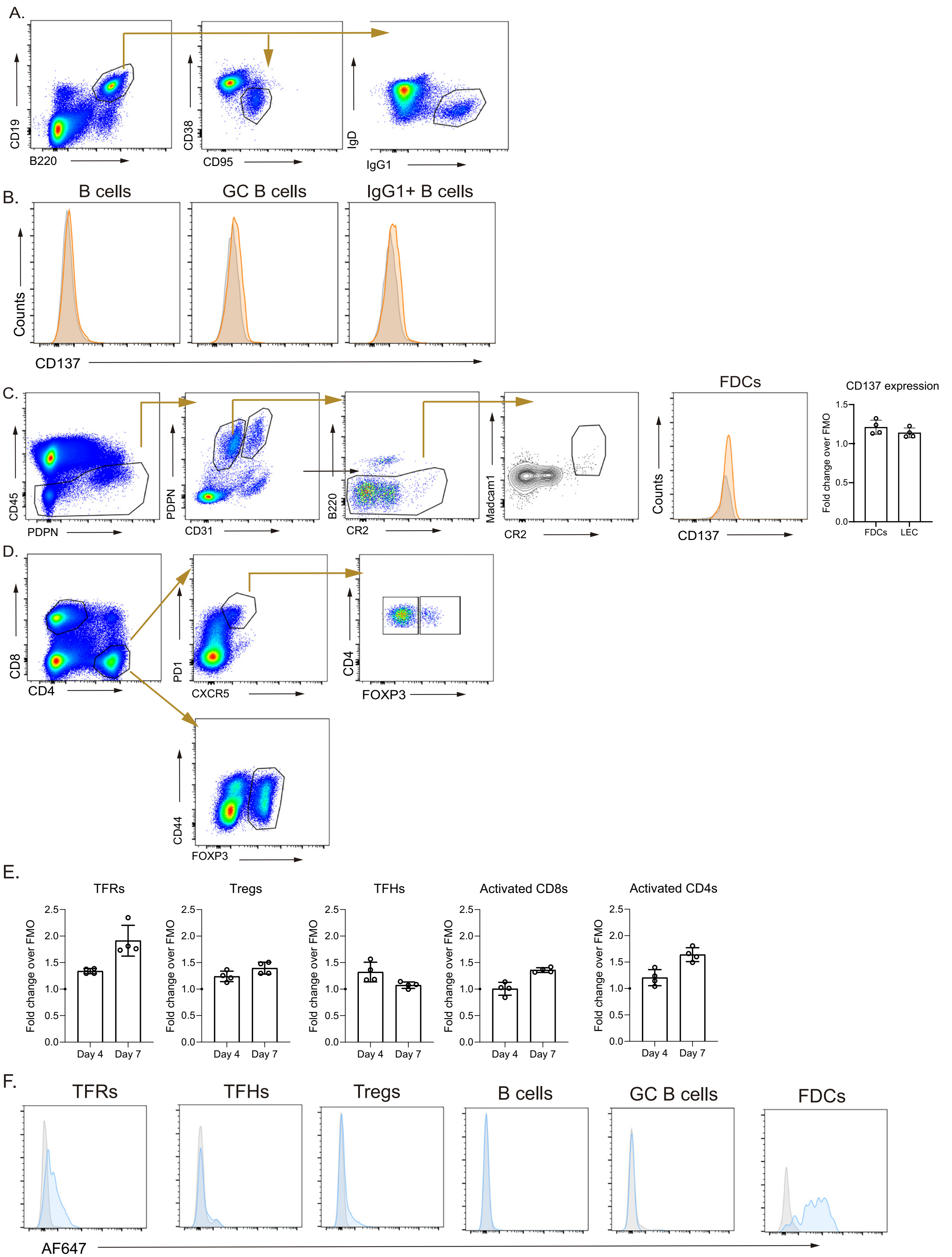
A.
CD19
B220
CD38
CD95
IgD
IgG1
B.
B cells
GC B cells
IgG1+ B cells
Counts
CD137
C.
CD45
PDPN
PDPN
CD31
B220
CR2
Madcam1
CR2
FDCs
Counts
CD137
CD137 expression
Fold change over FMO
FDCs
LEC
D.
CD8
CD4
PD1
CXCR5
CD4
FOXP3
CD44
FOXP3
E.
TFRs
Tregs
TFHs
Activated CD8s
Activated CD4s
Fold change over FMO
Day 4
Day 7
F.
TFRs
TFHs
Tregs
B cells
GC B cells
FDCs
AF647

Figure EV2. Analysis of CD137 expression and in vivo anti-CD137 targeting to lymphocyte populations and FDCs.

(**A**) Flow cytometry strategy to analyze B cells, GC B cells and IgG1 CS B cells. (**B**) Histograms of CD137 expression (orange) and FMO (gray) on total B cells, GC and IgG1^{+} B cells. (**C**) Gating strategy to analyze FDCs. CD137 expression (orange) and FMO (gray) on FDCs. Quantification of CD137 expression (MFI) in mice immunized with ovalbumin in Alhydrogel. Graph shows the fold change in MFI relative to normalized FMO controls ($n = 4$ mice; 1 independent experiment). (**D**) Gating strategy to select Tfh (CXCR5 + PD-1 +), Tfrs (CXCR5 + PD-1+ Foxp3+) and Tregs (CD4+ Foxp3+). (**E**) Quantification of CD137 expression (MFI) in mice immunized with ovalbumin in Alhydrogel for 4 and 7 days. Graph shows the fold change in MFI relative to normalized FMO controls (dotted line = 1) ($n = 4$ mice; 1 independent experiment). (**F**) Binding of AF647-labeled anti-CD137 (blue) and AF647-labeled IgG isotype (gray) to different dLNs immune populations. All quantitative data are shown as means ± SD.

 © The Author(s)

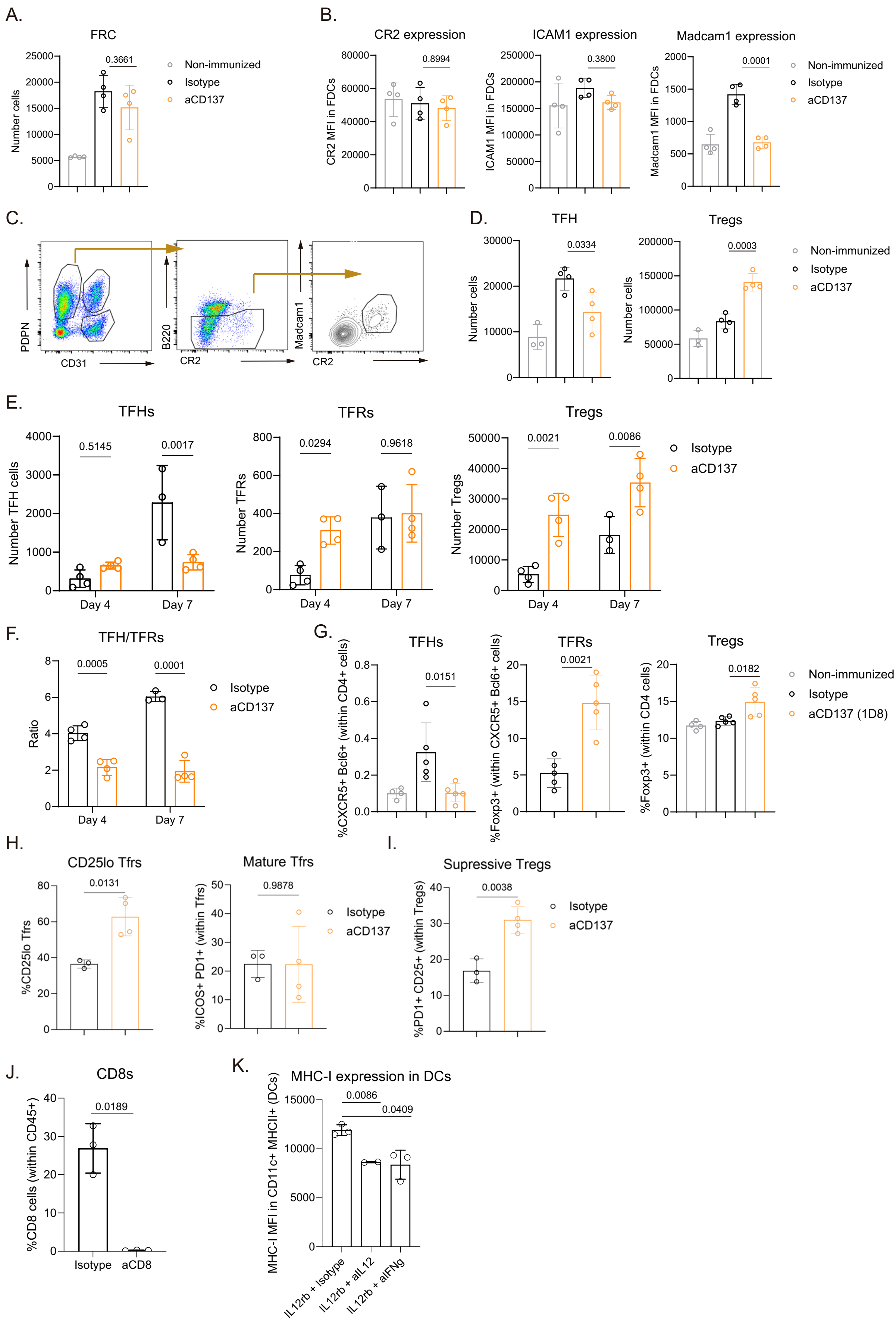
A.
FRC
Number cells
0.3661
Non-immunized
Isotype
aCD137
B.
CR2 expression
CR2 MFI in FDCs
0.8994
ICAM1 expression
ICAM1 MFI in FDCs
0.3800
Madcam1 expression
Madcam1 MFI in FDCs
0.0001
Non-immunized
Isotype
aCD137
C.
PDPN
CD31
B220
CR2
Madcam1
CR2
D.
TFH
Number cells
0.0334
Tregs
Number cells
0.0003
Non-immunized
Isotype
aCD137
E.
TFHs
Number TFH cells
0.5145
0.0017
Day 4
Day 7
TFRs
Number TFRs
0.0294
0.9618
Day 4
Day 7
Tregs
Number Tregs
0.0021
0.0086
Day 4
Day 7
Isotype
aCD137
F.
TFH/TFRs
Ratio
0.0005
0.0001
Day 4
Day 7
Isotype
aCD137
G.
TFHs
%CXCR5+ Bcl6+ (within CD4+ cells)
0.0151
TFRs
%Foxp3+ (within CXCR5+ Bcl6+ cells)
0.0021
Tregs
%Foxp3+ (within CD4 cells)
0.0182
Non-immunized
Isotype
aCD137 (1D8)
H.
CD25lo Tfrs
%CD25lo Tfrs
0.0131
Mature Tfrs
%ICOS+ PD1+ (within Tfrs)
0.9878
Isotype
aCD137
I.
Supressive Tregs
%PD1+ CD25+ (within Tregs)
0.0038
Isotype
aCD137
J.
CD8s
%CD8 cells (within CD45+)
0.0189
Isotype
aCD8
K.
MHC-I expression in DCs
MHC-I MFI in CD11c+ MHCII+ (DCs)
0.0086
0.0409
IL12rb + Isotype
IL12rb + aIL12
IL12rb + aIFNg

Figure EV3. CD137-agonism in vivo modifies Treg and Tfh numbers in a manner independent of CD8-T cells, IFNγ and IL12.

(**A**) Number of FRCs on the dLNs of non-immunized and immunized mice treated with anti-CD137 or its isotype-matched control. (**B**) Expression (MFI) of CR2, ICAM1, and MAdCAM1 on FDCs ($n = 4$ mice/group). (**C**) Gating strategy used to analyze FDCs on Fig. 3. (**D**) Number of Tfh and Treg cells on the dLNs of non-immunized and immunized mice treated with anti-CD137 or its isotype control ($n = 4$ mice/group). (**E**) Number of Tfhs, Tfrs and Tregs in immunized mice for 4 and 7 days treated with anti-CD137 or its isotype-matched control ($n = 3$–4 mice; 1 independent experiment). (**F**) Ratio Tfh/Tfrs at day 4 and 7 post-immunization ($n = 4$ mice). (**G**) Quantification of the percentage of Tfhs, Tfrs, and Tregs in non-immunized or immunized mice treated with anti-CD137 1D8 mAb or its isotype-matched control ($n = 5$ mice; 1 independent experiment). (**H**) Quantification of the percentage of Tfrs expressing low CD25 on the membrane (left graph) and ICOS + PD-1+ double-positive cells in mice immunized and treated with anti-CD137 or its isotype-matched control ($n = 3$–4 mice/group; 1 independent experiment). (**I**) Quantification of the percentage of PD-1 + CD25+ double-positive Tregs in mice treated as in (**H**). (**J**) Quantification of the percentage of CD8-T cells in the LNs of mice treated with the anti-CD8β depleting antibody or its isotype control ($n = 3$ mice/group; 1 independent experiment). (**K**) Quantification of the MFI of MHC-I expressed by DCs in mice injected with recombinant IL-12 (10 μg) and treated with neutralizing anti-IL12, anti-IFNγ or isotype control at days -1 and 1 ($n = 2$–3 mice/group; 1 independent experiment). All quantitative data are shown as mean ± SD and analyzed using one-way ANOVA with multiple comparisons or Welch's *t*-test.

© The Author(s)

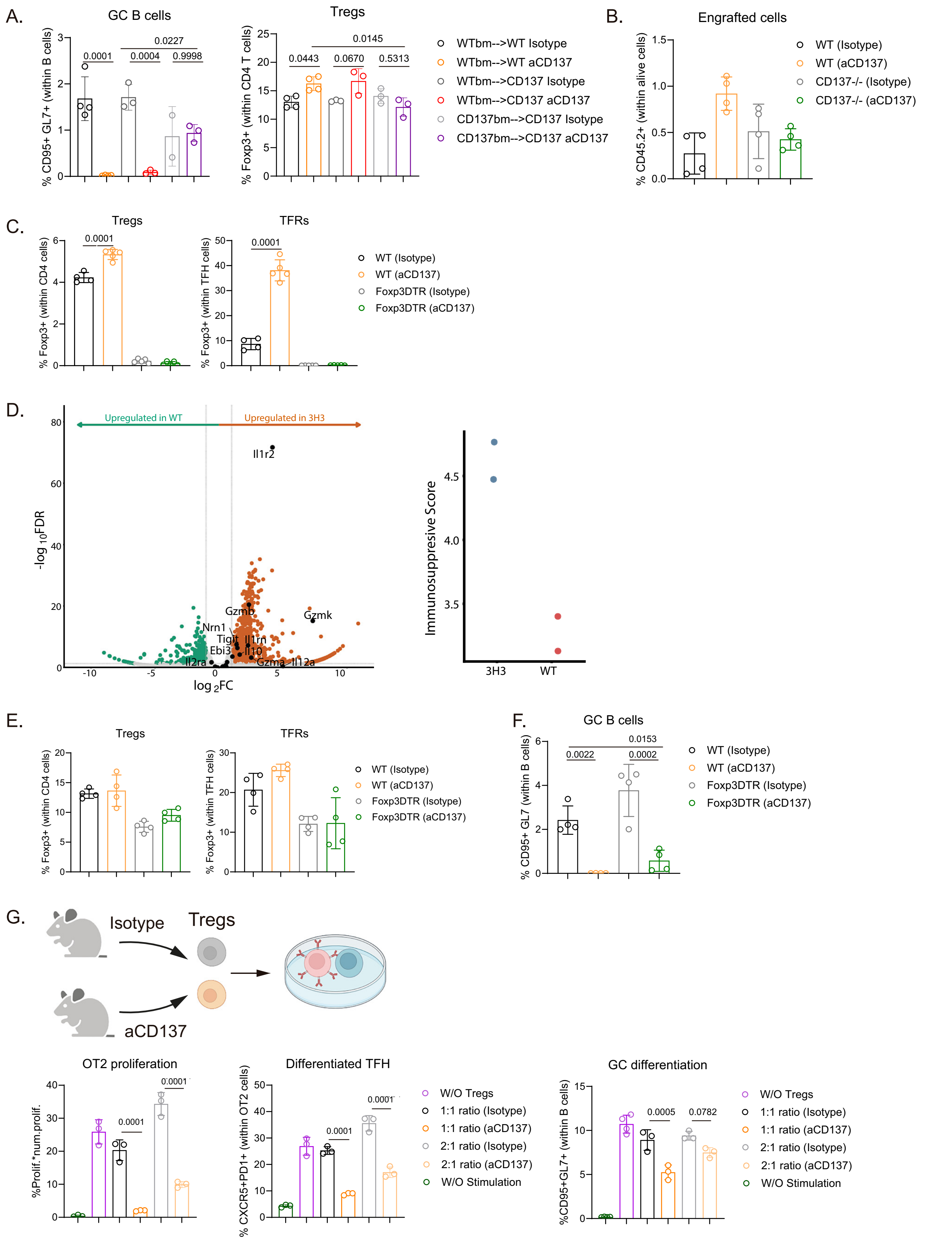
A.
GC B cells
% CD95+ GL7+ (within B cells)
0.0001
0.0227
0.0004
0.9998
Tregs
% Foxp3+ (within CD4 T cells)
0.0145
0.0443
0.0670
0.5313
WTbm-->WT Isotype
WTbm-->WT aCD137
WTbm-->CD137 Isotype
WTbm-->CD137 aCD137
CD137bm-->CD137 Isotype
CD137bm-->CD137 aCD137
B.
Engrafted cells
% CD45.2+ (within alive cells)
WT (Isotype)
WT (aCD137)
CD137-/- (Isotype)
CD137-/- (aCD137)
C.
Tregs
% Foxp3+ (within CD4 cells)
0.0001
TFRs
% Foxp3+ (within TFH cells)
0.0001
WT (Isotype)
WT (aCD137)
Foxp3DTR (Isotype)
Foxp3DTR (aCD137)
D.
Upregulated in WT
Upregulated in 3H3
-log 10FDR
log 2FC
Il1r2
Gzmb
Gzmk
Nrn1
Tigit
Il1rn
Ebi3
Il10
Il2ra
Gzma
Il12a
Immunosuppresive Score
3H3
WT
E.
Tregs
% Foxp3+ (within CD4 cells)
TFRs
% Foxp3+ (within TFH cells)
WT (Isotype)
WT (aCD137)
Foxp3DTR (Isotype)
Foxp3DTR (aCD137)
F.
GC B cells
% CD95+ GL7 (within B cells)
0.0153
0.0022
0.0002
WT (Isotype)
WT (aCD137)
Foxp3DTR (Isotype)
Foxp3DTR (aCD137)
G.
Isotype
Tregs
aCD137
OT2 proliferation
%Prolif.*num.prolif.
0.0001
0.0001
Differentiated TFH
% CXCR5+PD1+ (within OT2 cells)
0.0001
0.0001
W/O Tregs
1:1 ratio (Isotype)
1:1 ratio (aCD137)
2:1 ratio (Isotype)
2:1 ratio (aCD137)
W/O Stimulation
GC differentiation
%CD95+GL7+ (within B cells)
0.0005
0.0782
W/O Tregs
1:1 ratio (Isotype)
1:1 ratio (aCD137)
2:1 ratio (Isotype)
2:1 ratio (aCD137)
W/O Stimulation

Figure EV4. CD137-agonism acting in bone marrow-derived cells increases Treg/Tfr immunosuppressive activity.

(**A**) Percentage of GC B cells and Tregs in the indicated bone marrow reconstituted mice ($n = 2$–4 mice/group; 1 independent experiment). (**B**) Percentage of engrafted CD45.2+ cells on CD45.1+ host mice 14 days after immunization ($n = 4$ mice/group; 1 independent experiment). (**C**) Percentage of Tregs and Tfrs on the LNs of WT and Foxp3-DTR mice injected with anti-CD137 or its isotype and 660 ng of DTx ($n = 4$ mice/group; representative of two independent experiments). (**D**) Volcano plot showing the magnitude of the transcriptional changes (log2 fold change) together with the statistical significance (−log10 FDR) of the genes expressed by Tregs from isotype- or anti-CD137-treated mice. In black, highlight those genes that participate in the immunosuppressive gene score. The right graph shows the immunosuppressive score of the sequenced Foxp3+ cells. (**E**) Percentage of Tregs and Tfrs on the LNs of WT and Foxp3-DTR mice injected with anti-CD137 or its isotype and 66 ng of DTx ($n = 4$ mice/group; one independent experiment). (**F**) WT and Foxp3-DTR mice treated as in (**C**) but with 10× lower DTx dose (66 ng/dose) ($n = 4$ mice/group). (**G**) Co-culture assay workflow. Foxp3-GFP mice were injected with anti-CD137 or isotype control; 4 days later, GFP^+ cells were sorted and co-cultured for 3 days with purified B cells, CTV-labeled OT-II CD4 T cells, and 1 µm beads coated with anti-IgM $F(ab')_2$ and ovalbumin. Graphs show OT-II proliferation, as well as Tfh (CXCR5 + PD-1 +) and GC B cells (CD95 + GL7 +) percentages. Tregs were added at CD4 T:Treg ratios of 1:1 or 2:1; cultures without Tregs (magenta) and without bead stimulation (green) served as controls (representative of two independent experiments). All quantitative data were shown as means ± SD and analyzed using one-way ANOVA with multiple comparisons.

 © The Author(s)

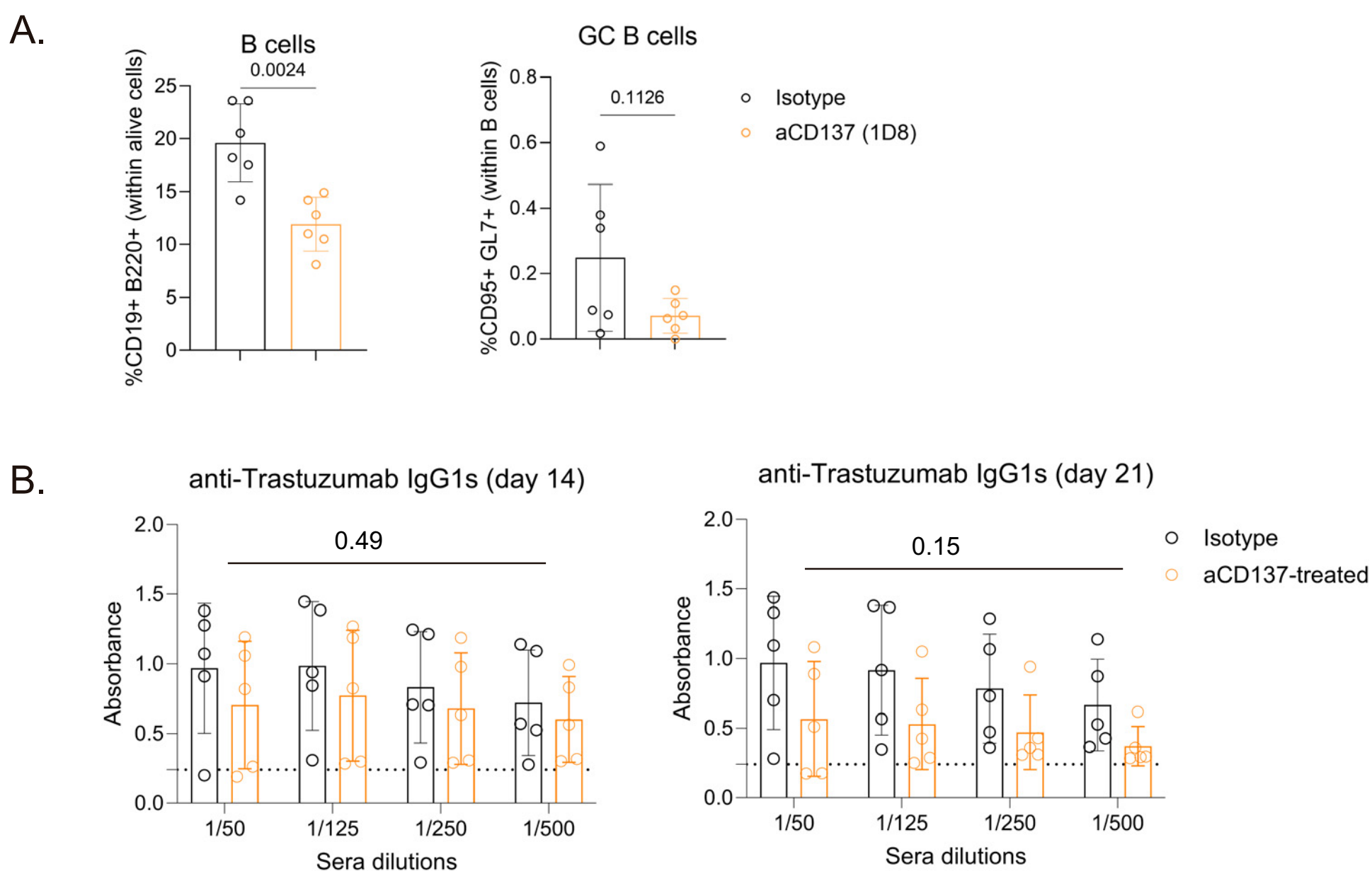

Figure EV5. Anti-CD137 disrupts tumor-associated TLS and ADA-associated humoral responses.

(**A**) Quantification of lung B cells and GC B cells in mice bearing KPAR tumors treated with isotype or anti-CD137 1D8 mAb ($n = 5$–6 mice/group; 1 independent experiment). (**B**) Absorbance levels of IgG1 anti-Trastuzumab antibodies in the sera of mice immunized with Trastuzumab and treated with isotype or anti-CD137 antibody after 14 days or 21 days post-immunization. The dotted line shows the background level of antibodies from non-immunized mice ($n = 5$ mice/group; 1 independent experiment). Quantitative data were shown as means ± SD and analyzed using two-way ANOVA or *t*-tests.

© The Author(s)
